# Integrated multiplex analysis of cell death regulators in stage II colorectal cancer suggests patients with ‘persister’ cell profiles fail to benefit from adjuvant chemotherapy

**DOI:** 10.1136/bmjonc-2024-000362

**Published:** 2024-08-06

**Authors:** Sanghee Cho, Elizabeth McDonough, John Graf, Jinru Shia, Canan Firat, Nil Urganci, Christine Surrette, Andreas Lindner, Manuela Salvucci, Anna Matveeva, Batuhan Kisakol, Anthony O’Grady, Mohammadreza Azimi, John P Burke, Deborah A McNamara, Simon McDade, Daniel B Longley, Jochen HM Prehn, Fiona Ginty

**Affiliations:** 1 Technology & Innovation Center, GE HealthCare, Niskayuna, NY, USA; 2 Department of Pathology, Memorial Sloan Kettering Cancer Center, New York, NY, USA; 3 Department of Physiology and Medical Physics, Royal College of Surgeons in Ireland University of Medicine and Health Sciences, Dublin, Ireland; 4 Centre for Systems Medicine, Royal College of Surgeons in Ireland University of Medicine and Health Sciences, Dublin, Ireland; 5 Department of Pathology, Royal College of Surgeons in Ireland University of Medicine and Health Sciences, Beaumont Hospital, Dublin, Ireland; 6 Department of Colorectal Surgery, Beaumont Hospital, Dublin, Ireland; 7 Patrick G Johnston Centre for Cancer Research, Queen's University, Belfast, UK

**Keywords:** Chemotherapy, Colorectal cancer, Tumour biomarkers

## Abstract

**Objective:**

Inducing tumour cell apoptosis is a primary objective of chemotherapy but, to date, there are no validated biomarkers of apoptosis sensitivity or resistance. Our objective was to image multiple apoptosis pathway proteins at single cell level and determine multi-protein associations with recurrence risk and chemotherapy response in patients with stage II colorectal cancer (CRC).

**Methods and analysis:**

Multiplexed imaging of 16 proteins in the intrinsic and extrinsic apoptosis pathways at single cell resolution on resected tissue from 194 patients with stage II CRC who either received adjuvant chemotherapy (n*=*108) or were treated with surgery only (n=86). K-means clustering of >600 000 cancer cells and cell level intensities of APAF1, procaspase-9, procaspase-3, XIAP, SMAC, BAX, BAK, BCL2, BCL-XL, MCL-1, procaspase-8, BID, FADD, FLIP, RIP3 and CIAP1 identified distinct cell cluster profiles.

**Results:**

Chemotherapy-treated patients with a higher percentage of cell clusters with low procaspase-3 and high XIAP had a higher risk of recurrence. This was validated in an independent cohort of adjuvant chemotherapy-treated high-risk patients with stage II CRC. We also applied two established system models of apoptosis initiation and execution to estimate cellular apoptosis sensitivity and show that these cell clusters do not appear to have impaired mitochondrial outer membrane permeabilisation sensitivity, but downstream procaspase-3 cleavage is compromised. This represents a key characteristic of drug-tolerant ‘persister’ cells.

**Conclusion:**

This study represents the most comprehensive analysis to date of apoptosis protein distribution at single cell level in CRC tumours. Our study identifies a subgroup of patients with stage II CRC with an apoptosis-resistant ‘persister’ cell profile who do not benefit from adjuvant chemotherapy.

**WHAT IS ALREADY KNOWN ON THIS TOPIC**

- A 2022 American Association of Clinical Oncology (ASCO) analysis showed that adjuvant chemotherapy is still prescribed for 20% of all patients with stage II colorectal cancer (CRC), the majority of whom are exposed to chemotherapy-induced adverse events with no real benefit.
- High-risk patients with stage II CRC are recommended to receive adjuvant chemotherapy, but studies have shown small effects on recurrence risk and survival.
- There are no approved predictive biomarkers of adjuvant chemotherapy response.

**WHAT THIS STUDY ADDS**

- Our goal was to determine whether tumour expression of multiple apoptosis proteins at single cell level in tumours of patients with stage II CRC predicted who would benefit or not from adjuvant chemotherapy.
- This is the first study to investigate the effects of multiple apoptosis proteins at single cell level.
- Previous studies analysed single markers and average or bulk protein expression and found mixed results.
- We demonstrate that only chemotherapy-treated patients with subpopulation of seemingly apoptosis-resistant ‘persister’ cell clusters have a higher risk of recurrence; increased recurrence risk was not found in patients undergoing surgery only.

**HOW THIS STUDY MIGHT AFFECT RESEARCH, PRACTICE OR POLICY**

- This study highlights the importance of multi-protein, single cell analysis to identify subpopulations of potentially apoptosis-resistant cancer cells, which may also be present in residual or micro metastatic disease post-surgery and thus contribute to chemotherapy resistance.
- More research is needed to validate a diagnostic testing approach for the detection and quantification of tumour ‘persister’ cells and to determine if patients with this cellular phenotype would benefit from apoptosis sensitisers.

## Introduction

 In 2020, colorectal cancer (CRC) accounted for 10% of global cancer incidence (1.9 million new cases) and 9.4% of cancer deaths (900 000).^1^ If diagnosed early (stage I) or where there is local spread and no lymph node involvement (stage II), the tumour is surgically removed, and the 5-year relative survival rates are 91% for colon and 90% for rectal cancer.^2^ High-risk patients with stage II CRC (ie, pT4 stage tumour or high-risk factors such as poor differentiation, inadequately sampled lymph nodes (<10–12) or histological signs of vascular, lymphatic or perineural invasion^3^) have a 5-year recurrence rate of 40–50%^3^ and are recommended to receive adjuvant chemotherapy with the anti-metabolite 5-fluorouracil (5-FU) or capecitabine (an oral prodrug of 5-FU).^3 4^ In high-risk patients with stage II CRC, 5-FU has been shown to decrease risk of death by only 3–5%.^5^,^7^ Conversely, many patients with stage II CRC with high recurrence risk are not treated with 5-FU-based chemotherapy, as they are considered ‘low risk’ based on pathological and histopathological staging. Identifying patients with stage II CRC who would benefit or not from adjuvant chemotherapy would greatly impact clinical practice.

Dysfunctional apoptosis is recognised as a key factor in tumour progression and development of chemotherapy resistance.^8^ At the molecular level, the apoptosis signalling network comprises a complex interplay of multiple activation pathways, inhibition and de-repression interactions, coupled with positive and negative feedback loops.^9 10^ The extrinsic pathway initiates with a pro-death signal originating from outside the cell.^11 12^ Intrinsic apoptosis is triggered by mitochondrial outer membrane permeabilisation (MOMP), which is regulated by oligomerisation of BAX and BAK, leading to the release of pro-apoptotic factors from the mitochondria. BAX/BAK oligomerisation is triggered by BH3-only proteins such as PUMA and NOXA (activated by DNA damage) and BID (activated by caspase-8 downstream of death receptors). Anti-apoptotic BCL2 family proteins, such as BCL2, BCL-XL and MCL-1, inhibit BH3-only proteins and BAX/BAK activation, thereby inhibiting MOMP.^13^ If MOMP is efficiently activated, the release of mitochondrial factors such as cytochrome-c and SMAC occurs, leading to the activation of initiator caspase-9 (activated by formation of the apoptosome containing cytochrome-c and APAF-1) and effector caspase-3 (the activation of which is enhanced by SMAC which neutralises the caspase-inhibitory activity of XIAP). Active caspase-3 executes the terminal cell death decision by cleaving hundreds of cellular substrates. The intrinsic and extrinsic pathways are connected via several proteins including XIAP, caspase-8 and BID, and both pathways converge on the activation of caspase-3.^9^ MOMP and caspase-3 activation have traditionally been considered to be ‘all-or-nothing’ processes; however, single cell imaging and molecular studies have identified the existence of ‘persister’ cells that may escape either sublethal MOMP or sublethal caspase-3 activation.^10 14 15^

To date, no single protein or group of proteins in the apoptosis pathway has yet been shown to reliably predict patient responsiveness to chemotherapy with the degree of sensitivity and specificity necessary to inform clinical decision-making.^16 17^ Our hypothesis was that analysis of multiple apoptosis and cell death pathway proteins at single cell level would account for apoptosis heterogeneity and provide a more sensitive predictor of chemotherapy benefit compared with single proteins. Using two independent cohorts with stage II CRC that were matched for risk factors but differed by treatment status, we posed the following questions: (1) Can disease recurrence in patients with stage II CRC cancer be predicted using an integrated single cell analysis of multiple apoptosis and cell death signalling proteins? (2) Is treatment response associated with the abundance of specific apoptotic signatures within the heterogeneous tumour epithelium? Our results showed that individual apoptosis proteins did not reveal significant or consistent correlations with recurrence risk. However, using K-means clustering of epithelial cells (>600 000) from across the patient cohorts, we identified independent cell clusters with distinct expression levels of proteins in the extrinsic and intrinsic pathways. We identified a cell cluster profile with higher-than-average expression of XIAP and lower in procaspase-3, which was correlated with higher risk of recurrence in chemotherapy-treated patients only. Using a dynamic system modelling approach, we demonstrated that this cluster profile contained cells exhibiting a ‘persister’ cell profile, characterised by a reduced sensitivity to apoptosome-dependent caspase activation. This was validated in an independent stage II CRC treated cohort.

## Results

### Cohort design and patient characteristics

**Figure 1:**
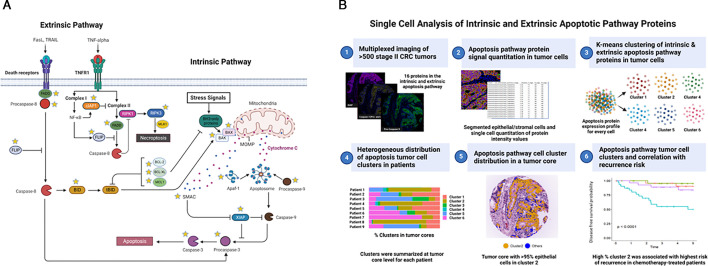
(A) Graphical representation of the proteins involved in the regulation of the extrinsic, intrinsic pathways and their roles in caspase activation, leading to apoptosis. Dye-conjugated antibodies for proteins highlighted with a yellow star were validated and underwent multiplexed staining and imaging (Cell DIVE) on tissue microarrays (TMAs) comprising of stage II CRC tumours. (B) End-to-end workflow for this study: (1) tissue microarrays constructed from surgery excised tumours underwent multiplexed imaging with 22 biomarkers using Cell DIVE (including 16 apoptosis pathway proteins highlighted in (A). Then, 194 closely matched patients were down-selected (n=86 underwent surgery alone/no chemotherapy; n=108 underwent surgery and adjuvant chemotherapy) for outcome analysis; (2) cells from the multiplexed images were segmented into epithelial and stromal cells and a spatial coordinate and ID was generated for every cell; signal intensity for each of the apoptosis pathway proteins was quantified for every cell; (3) tumour cells underwent K-means clustering of proteins in the intrinsic and extrinsic pathway combined; (4) cell clusters were aligned to each patient tumour and demonstrated heterogeneous distributions across patients; (5) cell clusters were mapped back to their spatial location in the tumour using cell ID and spatial coordinate. The example shown is a tumour with high percentage of cluster 2 cells (higher in XIAP, SMAC, CIAP1, lower in procaspase-3); (6) univariate and multivariable analysis was conducted for each cluster (the median was used as a cut-off) and associations with recurrence risk was determined in adjuvant chemotherapy-treated and surgery-only patients. The example Kaplan-Meier plot shows that cluster 2 from the combined intrinsic and extrinsic pathway analysis was associated with higher risk of recurrence in treated patients only (blue line). We further quantified apoptosis sensitivity of cluster 2 cells using systems models DR_MOMP and APOPTO-CELL and found that cluster 2 cells had average MOMP sensitivity but low caspase-3 activity. We then repeated steps 3–6 focusing on just the intrinsic pathway proteins and found that adjuvant chemotherapy-treated patients with a high % of cluster 2 tumour cells (XIAP, SMAC and lower in procaspase-3) had a higher risk of recurrence, and this was validated in an independent stage II CRC cohort. Created with BioRender.com.

The apoptosis pathway proteins under investigation in this study are highlighted in figure 1A and the end-to-end workflow for apoptosis protein analysis at single cell level and prognostic analysis is shown in figure 1B. To evaluate the potential of apoptosis signalling proteins as biomarkers of prognosis and therapy response, the ideal dataset would be a randomly designed clinical trial cohort. For this study, we combined two stage II CRC cohorts to mimic random selection and ensure a similar distribution of available risk factors in chemotherapy-treated patients and surgery-only patients. The first cohort (Huntsville; ‘HV’) was comprised of 238 patients, which included 98 patients with stage II CRC who received adjuvant chemotherapy.^18^ The second cohort (‘MSK1’; n=333) had 19 adjuvant chemotherapy-treated patients and the remaining patients with stage II CRC (n=281) were treated with surgery only. To ensure balance between the two cohorts, we evaluated (1) clinicopathological risk factors and (2) apoptosis protein distribution. Addressing the first point, there were proportionally more T3 patients among surgery-only patients in the ‘MSK1’ cohort; therefore, 196 patients were randomly filtered out to ensure balanced T3 numbers between the adjuvant chemotherapy and surgery-only groups (see online supplemental table 1 for clinical summary statistics before and after filtering and methods section; online supplemental figure 1 for graphical summary of clinical features of each cohort before and after filtering; online supplemental figures 2 and 3 for data flow-down, patient numbers at each step and final merged data set of adjuvant chemotherapy-treated or surgery only patients). Cellular intensity distribution for the apoptosis proteins was similar for both cohorts (online supplemental figure 4). The final combined cohort included 194 patients, 86 surgery-only and 108 adjuvant chemotherapy-treated, with no significant differences in clinical parameters between the two groups (online supplemental table 1). This study was performed in accordance with ethical guidelines for clinical research with the approval of the ethics committee for each institution. At Huntsville Clearview Cancer Center (IRB/CC1 Colon 01), all patients included in the study underwent surgery for histologically proven stage II CRC cancer between January 1997 and December 2008. For Memorial Sloan Kettering Cancer Center (IRB/WA0497-09), patients were selected who were surgically treated with curative intent for stage I and II CRC at Memorial Sloan Kettering Cancer Center between 1990 and 2010 (see the Materials and methods section).

### Multiplexed analysis of apoptosis proteins in the intrinsic and extrinsic pathways at single cell level

As highlighted in figure 1A, 16 proteins in the intrinsic apoptotic pathway (SMAC, XIAP, APAF1, procaspase-9, procaspase-3, MCL-1, BCL-XL, BCL2, BAX, BAK) and extrinsic apoptotic pathway (FADD, procaspase-3, procaspase-8, FLIP, RIP3, XIAP, CIAP1, BID) underwent multiplexed staining, imaging and single cell analysis on tumor tissue microarray (TMA) cores from each patient.^19^ Additional antibodies that were not successfully dye-conjugated (direct conjugation is required for highly multiplexed staining to prevent antibody cross-reactivity) included BIM, CIAP2, MLKL, RIPK1, TRAIL-R2. The final panel of proteins represents key components of both the pro-apoptotic and anti-apoptotic regulators in the intrinsic and extrinsic pathways, some of which are under investigation as potential therapeutic targets via direct and indirect inhibition or activation (previously extensively reviewed by Carneiro and El-Deiry^20^). For example, the BCL2 inhibitor venetoclax is approved for the treatment of several haematological malignancies, and the dual CIAP1/XIAP inhibitor tolinapant was granted orphan drug designation from the United States Food and Drugs Administration (FDA) for the treatment of T-cell lymphoma in 2020. Antibodies underwent a rigorous validation workflow^21^ (online supplemental table 2 for antibody details and the Materials and Methods section). TMAs for each cohort (n=8) were serially stained and imaged using Cell DIVE multiplexed immunofluorescence imaging (MxIF) workflow (as described in the Materials and methods section and in previous works^21 22^). Images underwent single cell segmentation (epithelial and stromal cells), and apoptosis protein staining intensities were quantified for every cell. The final total number of cells was 1 874 456 from 592 TMA cores and 194 patients, and of those, 685 140 epithelial cells, which we focused on for this study. Cell level correlations between the markers (in the combined datasets) are shown in online supplemental figure 5A. Highlighting the complexity of the apoptosis pathways and the interactions and competition between pro-apoptotic and anti-apoptotic proteins, many markers were positively correlated. The strongest correlations observed were between APAF1 (intrinsic pathway) and procaspase-8 (extrinsic; Spearman’s correlation coefficient rs=0.62), BCL-XL (intrinsic) and FADD (extrinsic; rs=0.63), and RIP3 (necroptosis) and FLIP (necroptosis and extrinsic apoptosis regulator; rs=0.63).

### Weak associations between average expression of single apoptosis pathway proteins and recurrence

To first benchmark against previously published immunohistochemistry analysis of single proteins,^9^ univariate analysis of average tumour cell intensity of the individual apoptosis proteins was conducted separately for the adjuvant chemotherapy-treated (‘treated’) and surgery without adjuvant chemotherapy (‘surgery only’) groups (online supplemental figure 5B). In surgery-only patients, none of the markers were significantly correlated with patient recurrence risk. In adjuvant chemotherapy-treated patients, markers associated with lower risk of recurrence, as assessed by calculating HRs, included higher RIP3, procaspase-8, procaspase-3, BCL2, APAF1, MCL-1; conversely, only high SMAC was associated with higher HR (higher risk of recurrence). However, after adjustment for multiple testing, none of the markers remained significant.

### Heterogeneity in apoptosis pathway protein expression at single cell level within and across patients

**Figure 2:**
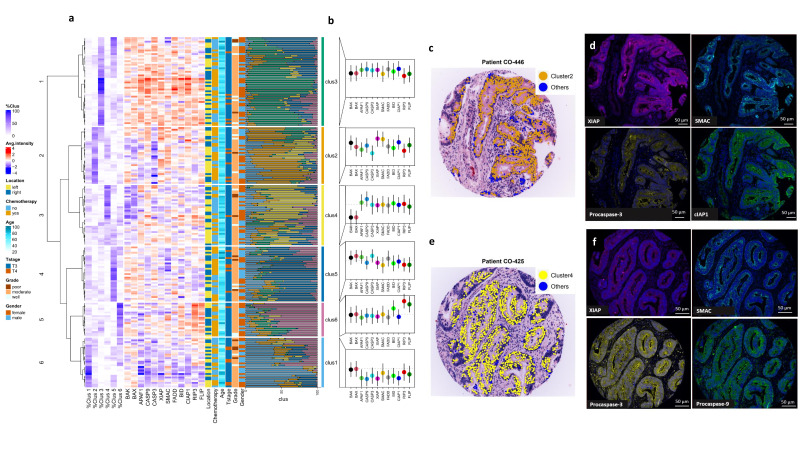
Proteins from the intrinsic and extrinsic pathway were combined for K-means cluster analysis to quantify protein distributions at a cellular level and contribution to tumour recurrence risk. (A) Integrated heatmap of clinical and protein data of all patients with stage II CRC from combined MSK1 and HV cohorts (adjuvant chemotherapy-treated and surgery only); cluster distribution for each patient is shown as a horizontal distribution of all six cluster groups; individual proteins, and clinical data (age, sex, tumour location, T stage, grade and treatment are included in the heat map for context); (B) lollipop plots of protein distributions in each cluster; (C) tumour with high % cluster 2 cells; (D) example staining features of this high cluster 2 tumour: high XIAP; high SMAC; low procaspase-3; high CIAP1. Scale bars 50 µm; (E) Tumour with high % cluster 4 cells; (F) example staining features of this high cluster 4 tumour: lower XIAP, average SMAC, high procaspase-9; average procaspase-3.

Recognising the need to consider the interdependencies and redundancies between apoptotic signalling proteins, we conducted K-means clustering of epithelial cells to determine whether there were repeated expression patterns of proteins in the intrinsic and extrinsic pathways (see analysis workflow in figure 1B) at a single cell level. This provided an unbiased assessment of apoptosis protein distribution and co-expression across all epithelial cancer cells. Given the known interrelationships and convergence points between the extrinsic and intrinsic pathways, we first clustered proteins from both pathways (‘integrated pathway’ approach) in all epithelial cells from adjuvant chemotherapy-treated and surgery-only patients. Proteins with the strongest contribution to cluster separation were down-selected using the Davies-Bouldin’s index^23^ and were comprised of BAX, BAK, APAF1, procaspase-9, procaspase-3, XIAP, SMAC, FADD, BID, CIAP1, RIP3 and FLIP. Using this approach, six distinct cell clusters were identified using consensus clustering. Marked heterogeneity in cluster distribution was noted across all patients (figure 2A). The lollipop distribution plots for each protein in each cluster are shown in figure 2B: cluster 1 cells had lower than average expression of most proteins; cluster 2 had higher than average XIAP, SMAC, CIAP1 and lower procaspase-3; cluster 3 had higher-than-average expression of all markers; cluster 4 had higher-than-average procaspase-9, low BAX and BAK; cluster 5 had higher procaspase-3, BAK, BAX, APAF1 and lower RIP3 and FLIP; cluster 6 had higher BID, RIP3 and FLIP. Figure 2C shows an example tumour image shown as a virtual hematoxylin and eosin (H&E) (pseudo-coloured DAPI and autofluorescence images) with cluster 2 cells highlighted in orange and example immunofluorescent images for XIAP, SMAC, procaspase-3 and CIAP1 (figure 2D); figure 2E shows a different tumour with a dominance of cluster 4 cells highlighted in yellow, along with example images for XIAP, SMAC, procaspase-3 and procaspase-9 (figure 2F). Online supplemental figure 6 shows UMAP cell clustering of all cells and cluster relationships, with highest separation between cluster 1 and cluster 3 cells.

### Cell clusters associated with increased recurrence risk in chemotherapy-treated patients

**Figure 3:**
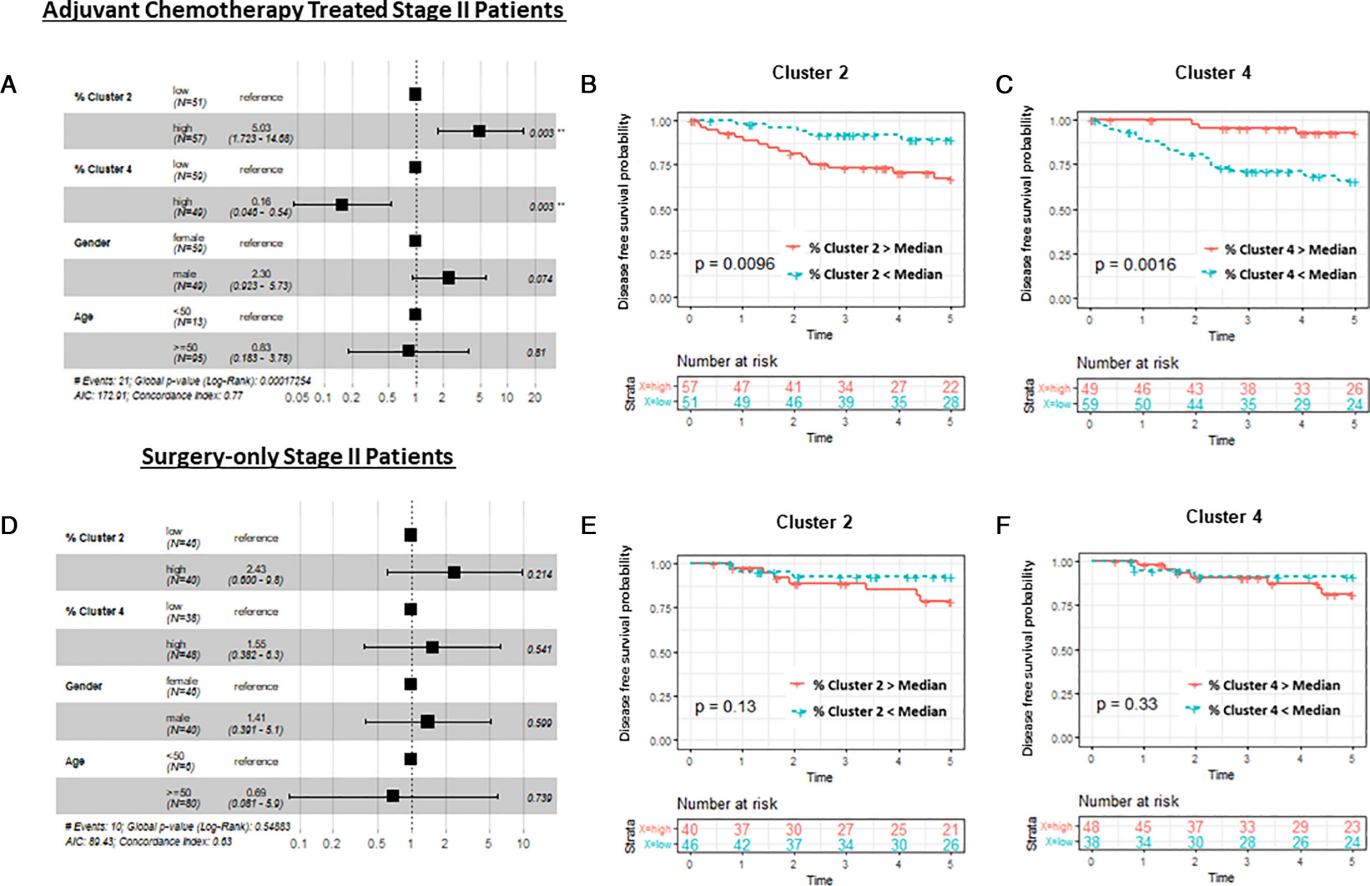
Cell clusters combining intrinsic and extrinsic pathway proteins and contribution to recurrence risk in stage II CRC patients. (A) Multivariable analysis adjusted for sex and age shows that adjuvant chemotherapy-treated patients with high % cluster 2 (low procaspase-3, high XIAP, SMAC, CIAP1) had a significant recurrence risk (p=0.003). Conversely patients with high % cluster 4 (higher in procaspase-9, APAF1 and lower in BAK and BAX) had a significantly lower recurrence risk. (B, C) Kaplan-Meier analysis of cluster 2 and cluster 4 in the same patients. (D–F) In surgery-only treated patients, no significant associations were found between cluster 2 or 4 (or any other cluster group) and recurrence risk.

In multivariable models adjusted for sex and age (figure 3A), tumours from adjuvant chemotherapy-treated patients with a higher % of cluster 2 cells (higher XIAP, SMAC, cIAP1 and lower procaspase-3) had significantly increased recurrence risk (p=0.003), whereas tumours with higher % of cluster 4 cells (higher procaspase-9, low BAX and BAK) had significantly lower recurrence risk (p=0.003) (overall model was p<0.0001; AIC=172.91 and concordance index 0.77). In agreement with this, Kaplan-Meier analysis showed that chemotherapy-treated patients with high % of cluster 2 cells or low % of cluster 4 cells had a significantly higher recurrence risk compared with all the other cell cluster groups (figure 3B,C). Importantly, in surgery-only patients, neither cluster 2 nor cluster 4 had an impact on recurrence risk (figure 3D–F), indicating that the clinical importance of these cell clusters is related to their impact on response to adjuvant chemotherapy.

### Alignment of clusters with models of MOMP sensitivity and caspase-3 activity

**Figure 4:**
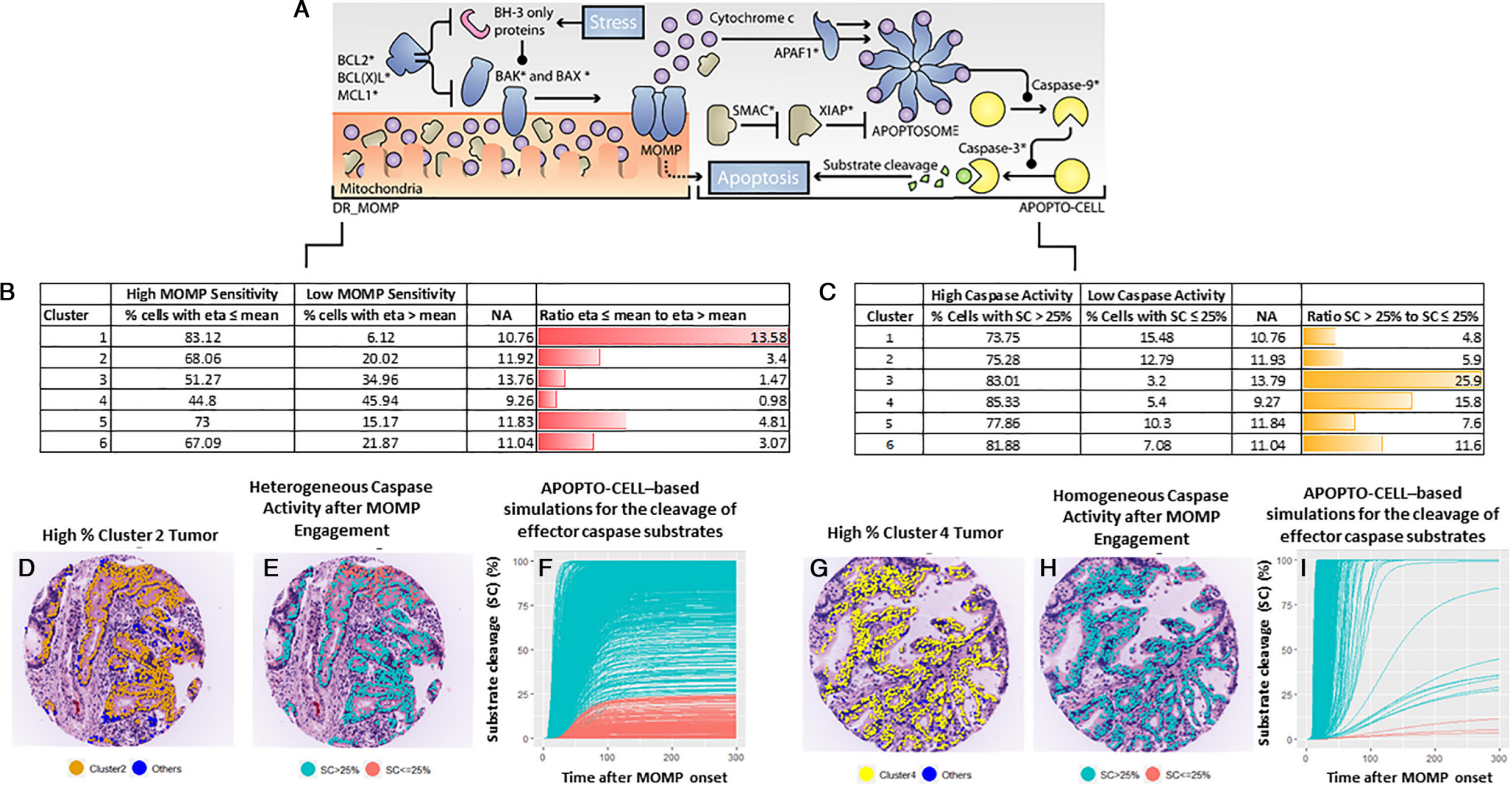
Modelling mitochondrial outer membrane permeabilisation (MOMP) sensitivity and caspase-3 cleavage and quantifying differences by cluster group—APOPTO-CELL systems model was used to calculate MOMP sensitivity and caspase 3 cleavage/activity for each cell. (A) The protein inputs (highlighted with *) for DR_MOMP (BCL2, BCL-XL, MCL, BAK and BAX) and APOPTO-CELL (APAF1, SMAC, XIAP, procaspase-3, procaspase-9). DR_MOMP eta metric is the maximum % of pores calculated after simulating an approximated mean stress dose. A low eta implies that cells can induce MOMP with a low amount of BH3 proteins and are hence more sensitive to stress and apoptosis. Eta ≤ or > population mean has been shown to discriminate between good and bad prognosis,^17^ and this cut-off was used for this study. For APOPTO-CELL model, substrate cleavage (SC) >25%, indicates high caspase-3 activity and ≤25%, indicates low caspase-3 activity. Low substrate cleavage has been previously demonstrated to be associated with chemotherapy resistance.^18 26 27^ (B) % cells with high MOMP sensitivity (eta≤mean) or low sensitivity (eta>mean) and ratio, aligned with each K-means cluster. Cluster 1 (all apoptosis pathway proteins were low with exception of average BAK and BAX) had the highest % of MOMP sensitive cells (83%) and the other clusters ranged from 50–70%, with cluster 2 (high in XIAP, SMAC, low in procaspase-3) having 68% MOMP sensitive cells. (C) % cells with high caspase activity (SC% >25%) and low caspase activity (SC% <25%), aligned with each K-means cluster. Cluster 4 (higher in procaspase 9, APAF1 and lower in BAK and BAX) had the highest % of cells with SC >25% (85.3% of cells) and cluster 1 (all markers were low except BAK and BAX) and cluster 2 had the lowest % cells with SC >25% (73.7% and 75.3%, respectively). (D–F) Example tumour sample dominant in cluster 2 cells and heterogeneous caspase-3 activity as determined by APOPTO-CELL model. (G–I) Tumour sample dominant in cluster 4 cells and uniformly high caspase-3 activity (APOPTO-CELL).

To interrogate the apoptosis sensitivity of the epithelial cells in clusters 2 and 4, we applied two established system models of apoptosis initiation and execution, the BCL2 pathway (DR_MOMP) and the caspase activation pathway (APOPTO-CELL). The DR_MOMP model (figure 4A) uses protein levels of BAK, BAX, BCL2, BCL-XL and MCL1 to determine a ‘stress dose’ or ‘eta’ required for MOMP for each cell^16 24 25^; cells with ≤10% mitochondrial pores are considered to have low sensitivity for MOMP. While the ≤10% pore cut-off has previously been experimentally validated and correlated with higher risk of recurrence in patients with stage III CRC,^16^ eta ≤ or > population mean has been shown to predict prognosis,^17^ and was used as the cut-off in this study. The APOPTO-CELL model uses cell protein levels of APAF1, procaspase-3, procaspase-9, SMAC and XIAP as inputs and models the cleavage and activation of caspase-3 downstream of MOMP.^10^ Using single cell imaging and caspase-3 FRET reporters, we previously demonstrated that using APOPTO-CELL, cells with a substrate cleavage (SC) ≤25% have inhibited caspase cleavage and fail to undergo apoptosis, whereas cells with SC>25% undergo caspase-3 cleavage/activation and apoptosis. APOPTO-CELL has been experimentally validated and shown to be prognostic in stage III CRC and glioblastoma.^1726^,^28^ We recently applied DR_MOMP and APOPTO-CELL models to spatially map cellular apoptosis competency in patients with stage III CRC and show significant inter-tumour and intra-tumour heterogeneity.^26^ Using the DR_MOMP model, the ratio of cells with high/low MOMP sensitivity in the high-risk cluster 2 group was 3.4 and close to 1 for low-risk cluster 4 (figure 4B); this suggests that cluster 2 cells have sensitivity to MOMP. However, using APOPTO-CELL, the ratio of cells with high/low caspase-3 cleavage was 5.9 in cluster 2 and 15.8 for cluster 4 (figure 4C). This indicates that while high-risk cluster 2 cells do not appear have impaired MOMP sensitivity, downstream caspase-3 cleavage/activation appears to be compromised. This represents a key characteristic of drug-tolerant ‘persister’ cells: in this case, cells that undergo MOMP, but survive because executioner caspase activation is below a threshold required for cell killing.^14^

### Intrinsic pathway proteins alone contribute to recurrence risk

To further investigate the critical proteins required for predicting recurrence risk, we repeated K-means clustering for proteins in the intrinsic (SMAC, XIAP, APAF1, procaspase-9, procaspase-3, MCL-1, BCL-XL, BCL2, BAX, BAK) or the extrinsic pathway (FADD, procaspase-3, procaspase-8, FLIP, RIP3, XIAP, CIAP1, BID) separately. Using the intrinsic pathway proteins, five distinct clusters were identified, which had similarities in protein distribution to the combined pathway clusters (online supplemental figure 7A). In multivariable analyses, a higher % of cluster 2 cells (higher XIAP, SMAC, lower procaspase-3) and lower % of cluster 4 cells (higher in procaspase-9) was significantly correlated with recurrence risk in chemotherapy-treated patients only (online supplemental figure 7B). This relationship was confirmed in Kaplan-Meier analyses (online supplemental figure 7C). Using the DR_MOMP and APOPTO-CELL system models, we found a similar MOMP sensitivity and caspase-3 cleavage profile to the integrated pathway model (ie, cluster 2 cells had average MOMP sensitivity and lower than average caspase-3 cleavage) (data not shown). None of the extrinsic pathway clusters identified were associated with recurrence risk in treated or surgery only patients (data not shown).

### Validation in an independent stage II cohort

**Figure 5:**
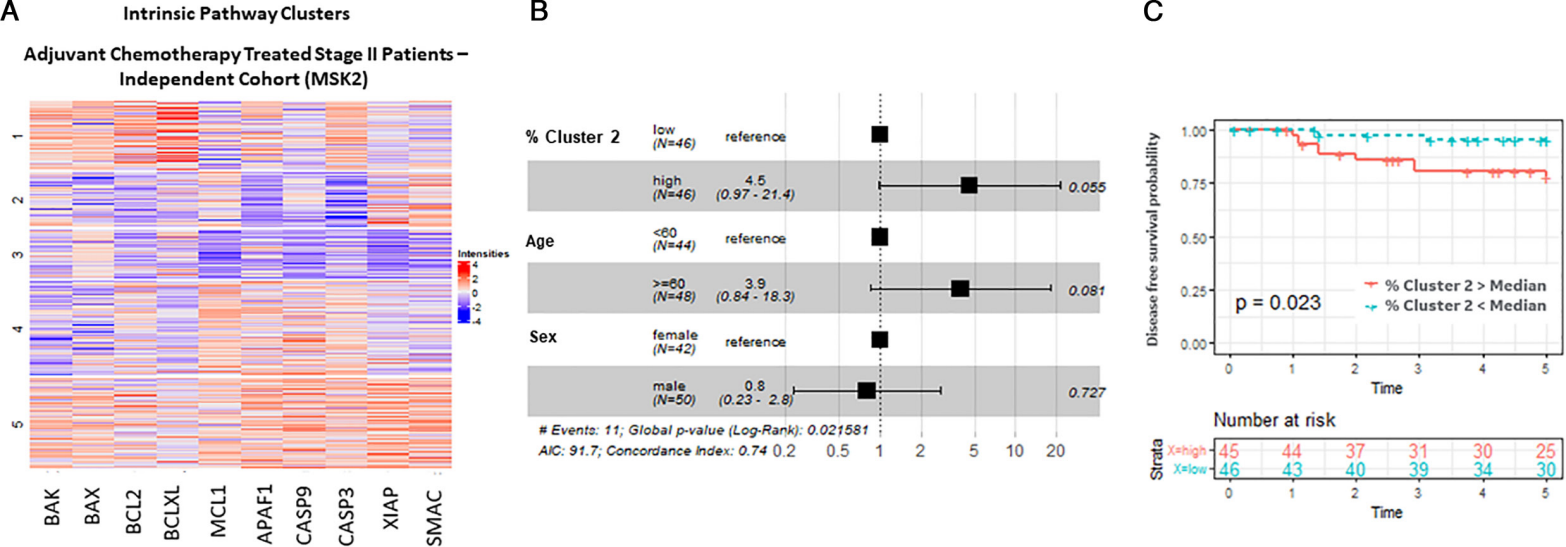
Intrinsic pathway protein cluster effects. We further investigated whether the proteins in the intrinsic pathway were driving recurrence risk and clustered proteins specific to this pathway (BAK, BAX, BCL2, BCLXL, MCL1, APAF1, procaspase-9, procaspase-3, XIAP and SMAC). We found a significant association with cluster 2 and recurrence risk in adjuvant chemotherapy treated patients in the HV and MSK1 cohorts (online supplemental figure 7). We repeated the analysis in an independent adjuvant chemotherapy-treated stage II CRC cohort (MSK2) shown here: (A) heatmap of cell clusters from the MSK2 cohort; (B) cluster 2 (lower procaspase-3, higher XIAP and SMAC) was significantly associated with recurrence risk in multivariable model (p=0.055) and in (C) Kaplan-Meier analysis (p=0.023).

We finally evaluated whether the intrinsic pathway clusters profiles were predictive in an independent cohort of patients with stage II CRC. Using a TMA from an adjuvant chemotherapy-treated stage II cohort that underwent multiplexed imaging and analysis using the same apoptosis pathway protein panel as the discovery cohort, we conducted K-means clustering of the intrinsic pathway proteins. This cohort (MSK2, n=91) consisted of more high-risk patients (see online supplemental table 3 and online supplemental figure 8), including more T4 patients (24% vs 13% in the HV/MSK1 treated cohort) and more poorly differentiated tumours (24% vs 7%), hence representing a selection of the ‘real world’ stage II CRC tumours treated with adjuvant chemotherapy. Here, we also found five distinct clusters with similar protein distributions using K-means clustering (figure 5A). A higher % of cluster 2 cells (higher XIAP and SMAC, lower in procaspase-3) was significantly associated with recurrence risk (p=0.023 and p=0.05 in multivariable model) (figure 5B,C). In this validation cohort, the high procaspase-9, low BAX/BAK cluster 4 group identified in the discovery cohort was less distinct, and no correlation with disease outcome was observed (data not shown). Again, using the DR_MOMP and APOPTO-CELL systems models, we found that cluster 2 cells had average MOMP sensitivity and lower than average caspase-3 cleavage (data not shown). In summary, these combined results suggest the existence of a population of micrometastatic ‘persister’ cells post-surgery that are characterised by a reduced sensitivity to apoptosome-dependent caspase activation, and that increase the risk of recurrence when exposed to chemotherapy. We further investigated the correlations between the significant clusters (ie, cluster 2 and 4 in the discovery cohort (HV and MSK1), cluster 2 in the validation cohort (MSK2) and clinical factors using Spearman’s correlation test for continuous variables, and Kruskal-Wallis test for categorical variables (online supplemental figure 9). Some of the clinical variables, including DNA mismatch repair (MMR), lymphovascular invasion, and local/distant metastasis, were only available for MSK1 and MSK2 patients. Although there were no significant differences after adjustment for multiple testing, there was a weak trend for MMR-proficient patients to have high % cluster 2 or high % cluster 4 cells. There was also a trend for female patients and T4 cancers to have higher % cluster 2 cells. We also evaluated associations between the clusters and local or distant metastases. Although non-significant after adjustment, MSK1 patients with high % cluster 4 tended to have more local recurrence than distant metastasis. Cluster 2 was not associated with local or distant metastases in MSK1 or MSK2 patients.

## Discussion

The benefit of adjuvant chemotherapy for patients with stage II CRC is debatable; therefore, better understanding of factors contributing to response are urgently needed to avoid unnecessary overtreatment and toxic side effects. Three significant clinical trials have been conducted in patients with stage II CRC, including the QUASAR clinical trial, which showed a 3.6% survival benefit within 2 years of surgery^27^; the National Surgical Adjuvant Breast and Bowel Project (NSABP; studies C-01 to C-04), which compared surgery alone to chemotherapy in stage II and stage III colon cancer and found that chemotherapy decreased risk of recurrence regardless of risk status^28^; and the phase III MOSAIC trial (Multicenter International Study of Oxaliplatin/5-FU/LV in the Adjuvant Treatment of Colon Cancer) showed a trend for better disease-free survival in patients with stage II colon cancer.^29^ In a meta-analysis,^30^ 5-year disease-free survival was found to be 81.4% for patients with stage II colon cancer who underwent resection without adjuvant chemotherapy and 79.3% for patients receiving adjuvant chemotherapy. Analysis of retrospective data from patients with stage II colon cancer from the US National Cancer Database showed higher overall survival in low-risk patients who received adjuvant chemotherapy.^31^ However, other studies have reported benefits of adjuvant chemotherapy in patients with stage II pT4 only.^5 6^ Recent guidelines now recommend treatment for high-risk patients^4^; however, 20% of *all* patients with stage II colon cancer still receive chemotherapy, exposing them to the negative side effects of chemotherapy, without the benefits.^3^ In the current study, we found that adjuvant chemotherapy-treated patients with stage II CRC (mix of pT3 and pT4) with a ‘persister’ cell cluster profile had a higher risk of recurrence. Notably, this was not observed in patients with who only underwent surgery. Moreover, this observation was validated in an independent chemotherapy-treated higher risk stage II CRC cohort (25% pT4 and 24% poorly differentiated tumours). This suggests that patients with pT3 or pT4 with a ‘persister’ cell profile do not benefit from adjuvant chemotherapy.

A review of past clinical studies correlating single apoptosis pathway proteins with recurrence concluded that a single marker does not adequately represent the complexity of cellular apoptosis heterogeneity, and a combination of markers is needed to represent an ‘apoptotic tumour profile’.^9^ Benchmarking against these single marker prognostic studies, we found mixed results for the 16 apoptosis pathway proteins under investigation. In univariate analysis, higher expressions of BCL2, procaspase-8, procaspase-3, MCL-1 and RIP3 were each significantly associated with reduced recurrence risk and SMAC was significantly correlated with increased risk treated patients only, but after adjustment for multiple testing they were no longer significant. Previous studies across several cancer types, including CRC have shown that higher SMAC expression is associated with better prognosis, attributed to its antagonising effects on inhibitor of apoptosis proteins (IAP).^32^,^35^ However, these studies were relatively small sample size, and none of the findings were validated in independent cohorts. A more recent study showed that low SMAC H-score (and BAK and BAX) was associated with longer progression-free survival in chemotherapy-treated patients with melanoma, and this was further verified with analysis of The Cancer Genome Atlas (TCGA) data.^36^ Our previous work on modelling XIAP and SMAC kinetics showed that only about 10% of SMAC binds to XIAP before onset of caspase cleavage and SMAC overexpression had minimal effects on cleavage, whereas overexpression of XIAP inhibited cleavage.^10^ Our findings for cell cluster 2 appear to agree with this model whereby higher SMAC expression may be simply acting as a bystander to higher XIAP, which itself inhibits caspase-3.

To address the limitations of single markers and average expression, we used K-means clustering to quantify the effects of different distributions of multiple apoptosis proteins within the intrinsic and extrinsic pathways at cell level. While most patients had one dominant cluster, marked intra-tumour and inter-tumour heterogeneity among the other clusters was evident. For example, in the high-risk/cluster 2 dominant tumours, cluster 1 (all apoptosis proteins were lower than average) was the second most dominant cluster, and both clusters 1 and 2 accounted for about 25–90% of all cells in high-risk patients. Even though clusters 4, 5 and 6 (which had more apoptosis sensitive profiles) were also present in these tumours, our findings suggest that a dominant ‘persister’ cell profile increases the risk of resistance to adjuvant chemotherapy, leading to eventual recurrence. We further investigated apoptosis sensitivity of these clusters using models of MOMP (DR_MOMP) and caspase-3 cleavage (APOPTO-CELL), which have been extensively validated in earlier works.^10 17 24 37 38^ We also recently applied both models to build a tumour atlas of apoptosis competency and showed marked inter-tumour and intra-tumour heterogeneity in MOMP sensitivity and caspase activation by cell type.^26^ Cluster 2 showed close to average MOMP sensitivity (ratio of cells with high/low MOMP sensitivity in the high-risk cluster 2 group was 3.4 vs an overall average of 4.5); however, the ratio of cells with caspase activation/caspase inhibition was lowest in clusters 1 and 2 compared with all other clusters (4.8 and 5.9, respectively, vs overall average of 11.9), suggesting a ‘caspase inhibited’ or ‘persister’ profile whereby cells undergo MOMP efficiently but fail to fully execute apoptosis because of low executioner caspase activation (due to a low procaspase-3/XIAP ratio). ‘Persister cells’ have been reported to undergo MOMP but still able to survive because executioner caspase activation is below the threshold required for cell killing.^14^ Sublethal caspase activation in these cells may lead to DNA damage through the activation of the endonuclease CAD (caspase-activated DNAse) and the accumulation of additional mutations in these cells. Our study also raises the question as to whether patients with high cluster 2 ‘persister’ cells would benefit from adjuvant therapy with apoptosis sensitisers. The BCL2 antagonist venetoclax is in use clinically, and MCL-1 and BCL-XL antagonists, as well as BAX/BAK activators, are in clinical or preclinical development. However, as indicated above, our data indicate that recurrence in adjuvant chemotherapy-treated patients is not linked to an inability to undergo MOMP. Rather, it suggests that executioner caspase activation may not be sufficiently activated due to high XIAP. Thus, IAP antagonists such as tolinapant may represent an interesting treatment option for these patients. We (DBL/SMcD) are currently exploring the clinical effectiveness of tolinapant in combination with FOLFOX in the advanced disease setting.

There are some limitations to this study in that it was conducted solely on TMA cores (1–2 mm) and not on whole tumour sections. While each patient had at least two tumour cores selected by a specialised gastrointestinal (GI) pathologist, and represented 3000–6000 tumour cells, this is a small fraction of a whole tumour section (which could be more than 1000 times that). However, the findings for cluster 2 were consistent in an independent cohort. Another limitation is that 10 markers in the intrinsic pathway (plus 3–4 additional cell segmentation markers) are required to achieve the level of cellular granularity and sensitivity needed to quantify subpopulations of cells with divergent apoptosis pathway protein expression. Since multiplexed imaging is not yet mainstream in the clinical diagnostic setting, it presents challenges for clinical implementation, at least in the short term. Therefore, we attempted to further reduce the number of markers and limited cell cluster analysis to XIAP, SMAC and procaspase-3 and XIAP and procaspase-3 alone. While a high XIAP/low procaspase-3 cluster group was evident (data not shown), it was not significant, and the best separation and prediction of recurrence risk was only evident using the entire panel of markers. Non-significant trends (after multiple testing correction) were found for low tumour expression of procaspase-3 (<median expression) and increased recurrence risk in chemotherapy-treated patients (in both the discovery and validation cohort). Therefore, a multimarker cell analysis seems to be most sensitive approach for identifying subpopulations of high-risk ‘persister’ cells. Ongoing advancements in multiplexed immunofluorescent imaging technologies (higher throughput, more markers per round, automated analysis) and clinical adoption will be required for multimarker/single cell assays to be made more broadly available. Our secondary analysis of clinical factors showed some interesting trends, including higher % cluster 2 cells in more advanced T4 cancers and lower % in MMR-deficient patients. Future analysis in larger sample sets is needed to support these findings.

In summary, this study represents the most comprehensive, integrated analysis to date of apoptosis protein distribution at single cell level in stage II CRC tumours and identifies a subgroup of patients with stage II CRC with an apoptosis resistant ‘persister’ cell profile who do not benefit from chemotherapy. Future studies are required to determine if patients with this profile benefit from apoptosis sensitisers.

## Materials and methods

### Patient cohorts

This study was performed in accordance with ethical guidelines for clinical research with the approval of the ethics committee for each institution. At Huntsville Clearview Cancer Center (IRB/CC1 Colon 01), all patients included in the study underwent surgery for histologically proven stage II CRC cancer between January 1997 and December 2008. For Memorial Sloan Kettering Cancer Center (IRB/WA0497-09), patients who were surgically treated at Memorial Sloan Kettering Cancer Center with curative intent for stage I and II CRC between 1990 and 2010 were selected. Only patients without neoadjuvant or adjuvant therapy were included, and only cases for which we had access to tissue blocks on the original resection and clinical follow-up information were included. Patients’ exclusion criteria were as follows: postoperative mortality within 30 days; a limited follow-up period of less than 3 years in cases without recurrence; synchronous multiple cancers; positive surgical margins; concomitant inflammatory bowel disease; and familial adenomatous polyposis or previous malignancy within 5 years. Because this was a retrospective study of archived samples and data, patients or the public were not involved in the design, or conduct, or reporting or dissemination plans of our research.

### Combining cohorts for integrated discovery analysis

To evaluate the association of biomarkers and cell clusters with outcomes (recurrence within 5 years) and treatment response, we carefully combined two cohorts (HV and MSK1) in a way that minimised the effects of bias as a confounding factor. We evaluated two aspects to ensure balance between the two cohorts: (1) balanced clinicopathological risk factors and (2) biomarker comparability. For clinicopathological risk factors, χ^2^ tests were performed to determine whether there was any dependency between the risk factors and treatment status for each cohort. Available risk factors included age, sex, tumour location, T stage, tumour differentiation and nodal count. There was a high number of T3 patients among untreated patients in the MSK cohort, and 197 patients were randomly filtered out to achieve a balance of T3 patients in each group. Second, biomarker intensity was evaluated between the two cohorts. We first used our validated normalisation procedure^39^ to minimise the batch effect among different slides. We also needed to be cautious about any bias derived from cohort differences. Therefore, to test whether cohort differences were significantly larger than slide differences within the cohorts, JSD (Jensen Shannon Divergence^40^) was used to measure the distance between the two distributions. This allows us not only to evaluate the mean difference but also measure the difference of the entire distribution. To compare the distances among the slides within a cohort versus across the two cohorts, we used the Wilcoxan test with multiple hypothesis testing adjustment using Benjamini & Hochberg^41^ method.

### Antibody validation and multiplexing workflow

All antibodies used in this study were subjected to a standardised characterisation process using a TMA and appropriate controls to evaluate the specificity and sensitivity of the primary antibody and its dye-conjugated derivative, including the cyclic testing of the dye inactivation treatment compared with singleplex staining. This protocol is described in detail on protocols.io^21^ and Gerdes *et al*.^19^ All markers and antibody clones and final concentrations are shown in online supplemental table 1. Multiplexed immunofluorescence (MxIF) staining of the skin samples was performed as previously described^19^ using Cell DIVE technology (Leica Microsystems, Issaquah, Washington, USA). After deparaffinisation and a two-step antigen retrieval, the formalin fixed paraffin embedded (FFPE) slides were stained with DAPI and imaged in all channels of interest to acquire background autofluorescence (AF) of the tissue. This was followed by indirect detection and/or direct conjugate antibody staining of up to three markers per round plus DAPI, dye deactivation and repeat staining to collect images of all planned biomarkers. Multiplexed images are automatically registered and processed for illumination correction and autofluorescence subtraction during each round of imaging using the Cell DIVE image processing workflow.

### Image and data processing

Cells in the epithelial and stromal compartments were segmented using DAPI, pan-cytokeratin, S6 and NaKATPase, as previously described.^19 22^ Images for every core were then reviewed for tissue quality (tissue loss or damage) and image segmentation quality. Images not passing the criteria (poor biomarker staining, too few cells or poor segmentation due to damage or poor staining of segmentation markers) were excluded from data analysis. Several quality control steps were conducted: (1) cell filtering based on the following criteria: epithelial cells required to have 1–2 number of nuclei; (2) each subcellular compartment (nucleus, membrane, cytoplasm) area>10 pixels<1500 pixels; and (3) cells in each round of staining have to have good alignment (minimum 80% for Huntsville cohort, and 85% for MSK cohorts) with first round of staining (automatic tissue quality index=1 at each round, which is the correlation between each image and the DAPI image). After quality review, the data were further processed including normalisation to remove batch effects,^39^ and log2 transformation to handle the skewness of the marker intensities.

### K-means clustering of intrinsic and extrinsic pathway proteins

We performed K-means clustering of markers in the intrinsic and extrinsic pathways to quantify the distributions of multiple apoptosis proteins at cell level. Extreme values (1% on both tails) were capped and standardised with zero mean and unit variance. Then, we applied K-means clustering with group size k=2,…,15. To determine the number of clusters that best represented the data, we also performed consensus clustering,^42^ then evaluated PAC (proportion of ambiguously clustered) and heatmaps. Once the number of groups was determined, a patient level cluster profile was calculated, which is the proportion of each cluster group within the entire cell population of a patient. The cluster profile at patient level was used to evaluate the correlation with the outcome (5-year recurrence) using Cox proportional model. Specifically, we performed the clustering in three sets of biomarkers: intrinsic and extrinsic pathway combined, and the intrinsic extrinsic pathways separately. For the integrated pathway model, we down-selected the markers using Davies-Bouldin’s index^23^ to measure the contribution of each individual markers to the cluster separation from the rest of the cells. To evaluate the cohort bias, we also performed K-means clustering after scaling within the cohort separately.

### DR_MOMP ODE model to calculate MOMP sensitivity

Protein levels of BAK, BAX, BCL2, BCL-XL and MCL1 were normalised to the mean protein levels in HeLa cells spotted on separate slides that were stained alongside slides used in this study. Protein molar concentrations were calculated using previously established HeLa concentrations and used as input for DR_MOMP as previously described.^24^ DR_MOMP was translated from its MATLAB implementation to C++ and R using deSolve (V.1.28), doParallel (V.1.0.15) and Rcpp (V.1.0.5). For each core, the maximum % level of pores was calculated after simulating an approximated mean stress dose (200 nM; estimated from the patient population as a threshold).^16 24 25^ Cells were considered to have low sensitivity for MOMP if the % level of pores was <10%.^24^ We found a Pearson’s correlation coefficient of −0.82 (95% CI −0.83 to −0.82; p < 0.001) between the % level of pores and the (previously) used stress dose required for MOMP (log-transformed) calculated for 10 000 randomly chosen cancer cells.^24^

### APOPTO-CELL model to calculate caspase-3 cleavage

The APOPTO-CELL model^10^ was implemented in the MATLAB environment equipped with Statistics and Parallel toolboxes (V.2014b, The MathWorks, Natick, Massachusetts, USA). Protein concentrations to use as input for the APOPTO-CELL model, namely, procaspase-3, procaspase-9, SMAC and XIAP, were estimated by aligning the signal intensities in arbitrary units to molar concentrations (in μM) with an established pipeline^17^ using as a reference protein molar profiles determined in a clinically relevant CRC cohort,^43^ as previously described.^17 26 38^ Previous research^38 44^ has shown APAF1 not to be the limiting factor in apoptosome formation in the CRC settings. Thus, APAF1 cell-specific protein levels were set to the median expression (0.123 μM) previously determined in clinically relevant CRC cohort,^44^ as previously described.^17 26 38^

### Statistical analysis

Univariate analysis was performed at patient level correlating the outcome and risk of recurrence within 5 years. For intrinsic and extrinsic pathway markers, we calculated the average intensity of all cells in the entire epithelial mask per patient, including across multiple cores for the same patient, when available. After binarising the continuous scale average using median cut point, Cox proportional hazard model was fitted for each biomarker on treated and untreated patients separately. Multiple hypothesis test correction using Benjamini & Hochberg^41^ method was applied. Inter-marker correlations were evaluated at cell level as well as a patient level (using average values), which was used for data quality control (QC) and to confirm expected biomarker associations. Following univariate analysis, stepwise feature selection (My.stepwise.coxph function in R) was used to optimise the Cox proportional hazard model performance.

### Patient and public involvement

As this was a retrospective study of archived samples and data, patients or the public were not involved in the design, or conduct, or reporting or dissemination plans of our research.

## Supplementary material

10.1136/bmjonc-2024-000362 online supplemental file 1

## Data Availability

Data are available upon reasonable request.

## References

[1] Xi Y, Xu P (2021). Global colorectal cancer burden in 2020 and projections to 2040. Transl Oncol.

[2] American Cancer Society Colorectal cancer survival rates | colorectal cancer prognosis. https://www.cancer.org/cancer/colon-rectal-cancer/detection-diagnosis-staging/survival-rates.html.

[3] Baxter NN, Kennedy EB, Bergsland E (2022). Adjuvant therapy for stage ii colon cancer: ASCO guideline update. J Clin Oncol.

[4] Argilés G, Tabernero J, Labianca R (2020). Localised colon cancer: ESMO clinical practice guidelines for diagnosis, treatment and follow-up. Ann Oncol.

[5] Kumar A, Kennecke HF, Renouf DJ (2015). Adjuvant chemotherapy use and outcomes of patients with high-risk versus low-risk stage II colon cancer. Cancer.

[6] Verhoeff SR, van Erning FN, Lemmens VEPP (2016). Adjuvant chemotherapy is not associated with improved survival for all high-risk factors in stage II colon cancer. Int J Cancer.

[7] Babcock BD, Aljehani MA, Jabo B (2018). High-risk stage II colon cancer: not all risks are created equal. Ann Surg Oncol.

[8] Hanahan D, Weinberg RA (2011). Hallmarks of cancer: the next generation. Cell.

[9] Zeestraten ECM, Benard A, Reimers MS (2013). The prognostic value of the apoptosis pathway in colorectal cancer: a review of the literature on biomarkers identified by immunohistochemistry. Biomark Cancer.

[10] Rehm M, Huber HJ, Dussmann H (2006). Systems analysis of effector caspase activation and its control by X-linked inhibitor of apoptosis protein. EMBO J.

[11] Wilson TR, McLaughlin KM, McEwan M (2007). C-FLIP: a key regulator of colorectal cancer cell death. Cancer Res.

[12] Walczak H, Krammer PH (2000). The CD95 (APO-1/fas) and the TRAIL (APO-2L) apoptosis systems. Exp Cell Res.

[13] Czabotar PE, Lessene G, Strasser A (2014). Control of apoptosis by the BCL-2 protein family: implications for physiology and therapy. Nat Rev Mol Cell Biol.

[14] Kalkavan H, Chen MJ, Crawford JC (2022). Sublethal cytochrome c release generates drug-tolerant persister cells. Cell.

[15] Giampazolias E, Zunino B, Dhayade S (2017). Mitochondrial permeabilization engages NF-κB-dependent anti-tumour activity under caspasedeficiency. Nat Cell Biol.

[16] Lindner AU, Salvucci M, Morgan C (2017). BCL-2 system analysis identifies high-risk colorectal cancer patients. Gut.

[17] Salvucci M, Würstle ML, Morgan C (2017). A stepwise integrated approach to personalized risk predictions in stage III colorectal cancer. Clin Cancer Res.

[18] Murphy CC, Harlan LC, Lund JL (2015). Patterns of colorectal cancer care in the United States: 1990-2010. J Natl Cancer Inst.

[19] Gerdes MJ, Sevinsky CJ, Sood A (2013). Highly multiplexed single-cell analysis of formalin-fixed, paraffin-embedded cancer tissue. Proc Natl Acad Sci U S A.

[20] Carneiro BA, El-Deiry WS (2020). Targeting apoptosis in cancer therapy. Nat Rev Clin Oncol.

[21] McDonough L, Chadwick C, Ginty F (2020). Protocols.io: cell DIVE^TM^ platform | antibody staining & imaging.

[22] Gerdes MJ, Gökmen-Polar Y, Sui Y (2018). Single-cell heterogeneity in ductal carcinoma in situ of breast. Mod Pathol.

[23] Davies DL, Bouldin DW (1979). A cluster separation measure. IEEE Trans Pattern Anal Mach Intell.

[24] Lindner AU, Concannon CG, Boukes GJ (2013). Systems analysis of BCL2 protein family interactions establishes a model to predict responses to chemotherapy. Cancer Res.

[25] Flanagan L, Lindner AU, de Chaumont C (2015). BCL2 protein signalling determines acute responses to neoadjuvant chemoradiotherapy in rectal cancer. J Mol Med (Berl).

[26] Lindner AU, Salvucci M, McDonough E (2022). An atlas of inter- and intra-tumor heterogeneity of apoptosis competency in colorectal cancer tissue at single-cell resolution. Cell Death Differ.

[27] Quasar Collaborative Group (2007). Adjuvant chemotherapy versus observation in patients with colorectal cancer: a randomised study. Lancet.

[28] Mamounas E, Wieand S, Wolmark N (1999). Comparative efficacy of adjuvant chemotherapy in patients with dukes’ B versus dukes’ C colon cancer: results from four national surgical adjuvant breast and bowel project adjuvant studies (C-01, C-02, C-03, and C-04). J Clin Oncol.

[29] André T, Boni C, Mounedji-Boudiaf L (2004). Oxaliplatin, fluorouracil, and leucovorin as adjuvant treatment for colon cancer. N Engl J Med.

[30] Böckelman C, Engelmann BE, Kaprio T (2015). Risk of recurrence in patients with colon cancer stage II and III: a systematic review and meta-analysis of recent literature. Acta Oncol.

[31] Reif de Paula T, Gorroochurn P, Simon HL (2022). A national evaluation of the use and survival impact of adjuvant chemotherapy in stage II colon cancer from the national cancer database. Colorectal Dis.

[32] Endo K, Kohnoe S, Watanabe A (2009). Clinical significance of Smac/DIABLO expression in colorectal cancer. Oncol Rep.

[33] Mizutani Y, Nakanishi H, Yamamoto K (2005). Downregulation of Smac/DIABLO expression in renal cell carcinoma and its prognostic significance. J Clin Oncol.

[34] Dai C-H, Li J, Shi S-B (2010). Survivin and Smac gene expressions but not livin are predictors of prognosis in non-small cell lung cancer patients treated with adjuvant chemotherapy following surgery. Jpn J Clin Oncol.

[35] Yan H, Yu J, Wang R (2012). Prognostic value of Smac expression in rectal cancer patients treated with neoadjuvant therapy. Med Oncol.

[36] Guttà C, Rahman A, Aura C (2020). Low expression of pro-apoptotic proteins bax, bak and Smac indicates prolonged progression-free survival in chemotherapy-treated metastatic melanoma. Cell Death Dis.

[37] Murphy ÁC, Weyhenmeyer B, Schmid J (2013). Activation of executioner caspases is a predictor of progression-free survival in glioblastoma patients: a systems medicine approach. Cell Death Dis.

[38] Salvucci M, Zakaria Z, Carberry S (2019). System-based approaches as prognostic tools for glioblastoma. BMC Cancer.

[39] Graf J, Cho S, McDonough E (2022). FLINO: a new method for immunofluorescence bioimage normalization. Bioinformatics.

[40] Lin JD (1991). Divergence measures based on the shannon entropy. IEEE Trans Inform Theory.

[41] Benjamini Y, Hochberg Y (1995). Controlling the false discovery rate: a practical and powerful approach to multiple testing. J R Stat Soc B.

[42] Wilkerson MD, Hayes DN (2010). ConsensusClusterPlus: a class discovery tool with confidence assessments and item tracking. Bioinformatics.

[43] Hector S, Prehn JHM (2009). Apoptosis signaling proteins as prognostic biomarkers in colorectal cancer: a review. *Biochim Biophys Acta*.

[44] Hector S, Conlon S, Schmid J (2012). Apoptosome-dependent caspase activation proteins as prognostic markers in stage II and III colorectal cancer. Br J Cancer.

