# Supplementary material for: Integrated multiplex analysis of cell death regulators in stage II colorectal cancer suggests patients with ‘persister’ cell profiles fail to benefit from adjuvant chemotherapy
Source: BMJ Oncol. 2024 Aug 6;3(1):e000362. doi: 10.1136/bmjonc-2024-000362 (PMC11347685; doi:10.1136/bmjonc-2024-000362)
Supplement: online supplemental file 1 [file bmjonc-2024-000362supp001.pdf]

Supplementary Figures

Supplementary Figure 1 a – Distribution of clinical parameters in HV and MSK1 stage II cohorts (chemotherapy untreated = no; treated = yes) before data filtering. In the original unfiltered cohort, HV was comprised of 238 patients which included 98 stage II patients who received adjuvant Chemotherapy (58 patients got 5-FU based therapy, and regimen was not specified for remaining 40 patients). The second cohort (“MSK1”; n=333) had 19 treated patients and the most of the remaining stage II patients (n=281) were treated by surgery only. The two cohorts were combined in a way that minimized the effects of bias on the analysis. Proportionately, there were more T3 patients among untreated patients in the “MSK1” cohort; 196 of those were randomly filtered out to ensure balanced T3 numbers between the chemotherapy treated and untreated patients. Patients with the highest nodal count were also excluded; **b -** after patient filtering, the combined cohort included 194 patients, 86 untreated and 108 treated, with no significant differences in clinical parameters between the two groups.

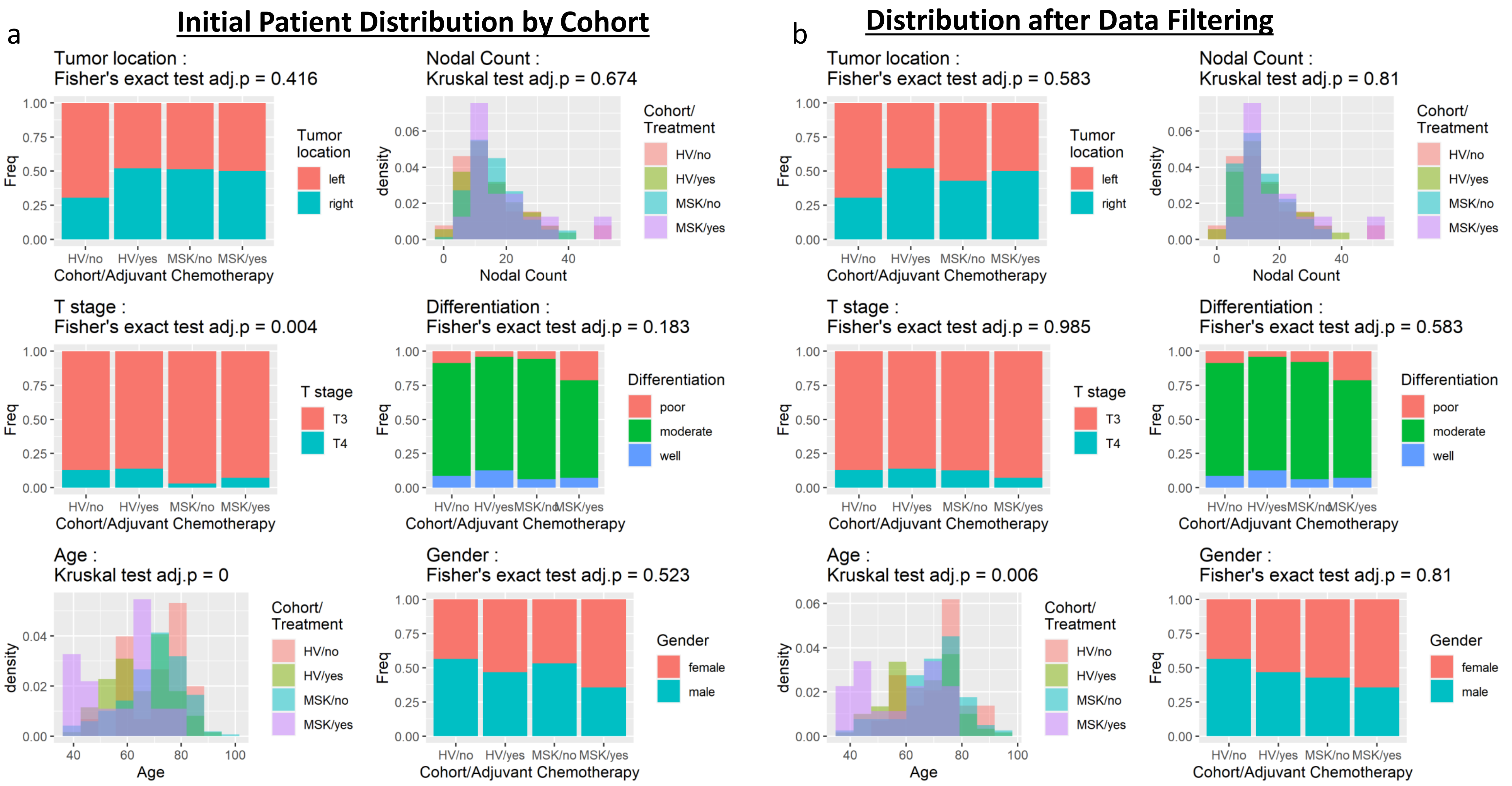

Supplementary Figure 2: Flow-down chart for each step of data filtering, including clinical parameters, data QC and final merger of stage II HV and MSK1 cohorts (n=194 patients);

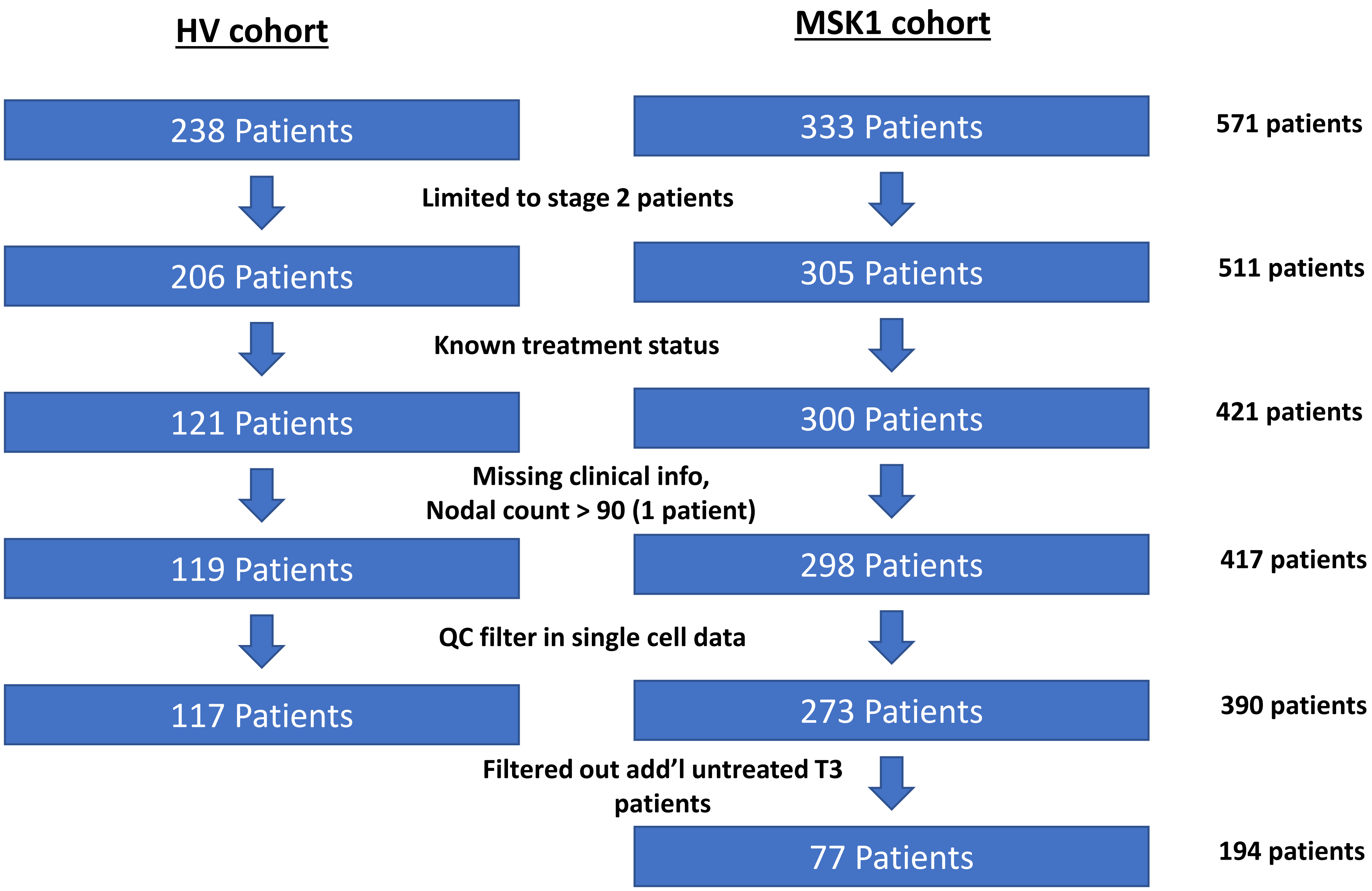

Supplementary Figure 3: a - Following segmentation of cells for each TMA core, there are a number of QC steps to ensure only high quality processed single cell data is included. This flow down chart provides additional information on the number of cell-like objects that we started with and the final number of cells that fulfilled QC criteria: 1) epithelial cells required to have 1-2 number of nuclei; 2) each sub-cellular compartment (nucleus, membrane, cytoplasm) area > 10 pixels < 1500 pixels; and 3) cells in each round of staining have to have good alignment (minimum 80% for Huntsville cohort, and 85% for MSK cohort) with first round of staining (automatic tissue quality index=1 at each round, which is the correlation between each image and the DAPI image); **b -** Final distribution of patients matched by risk factor and clinical demographic data in the merged untreated and treated groups.

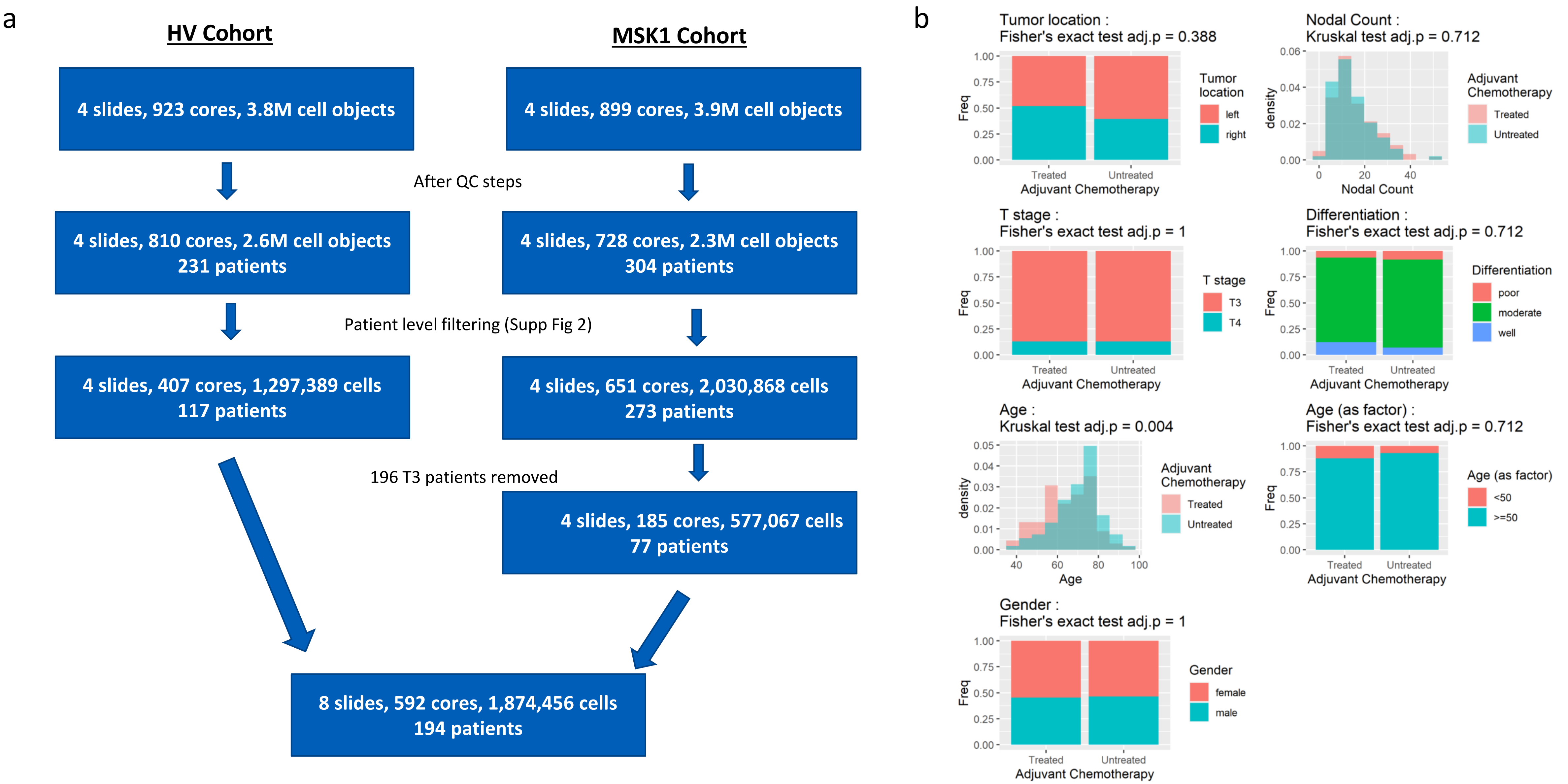

Supplementary Figure 4 – After the treated and untreated patients were down-selected for analysis (n=194), the mean cell intensities for each biomarker were compared across 8 TMA slides and by cohort. Distributions for all 16 apoptosis markers for each slide and color coded by cohort are shown. No significant differences in cell protein levels were found between slides and cohorts which made it feasible to combine the data from both cohorts.

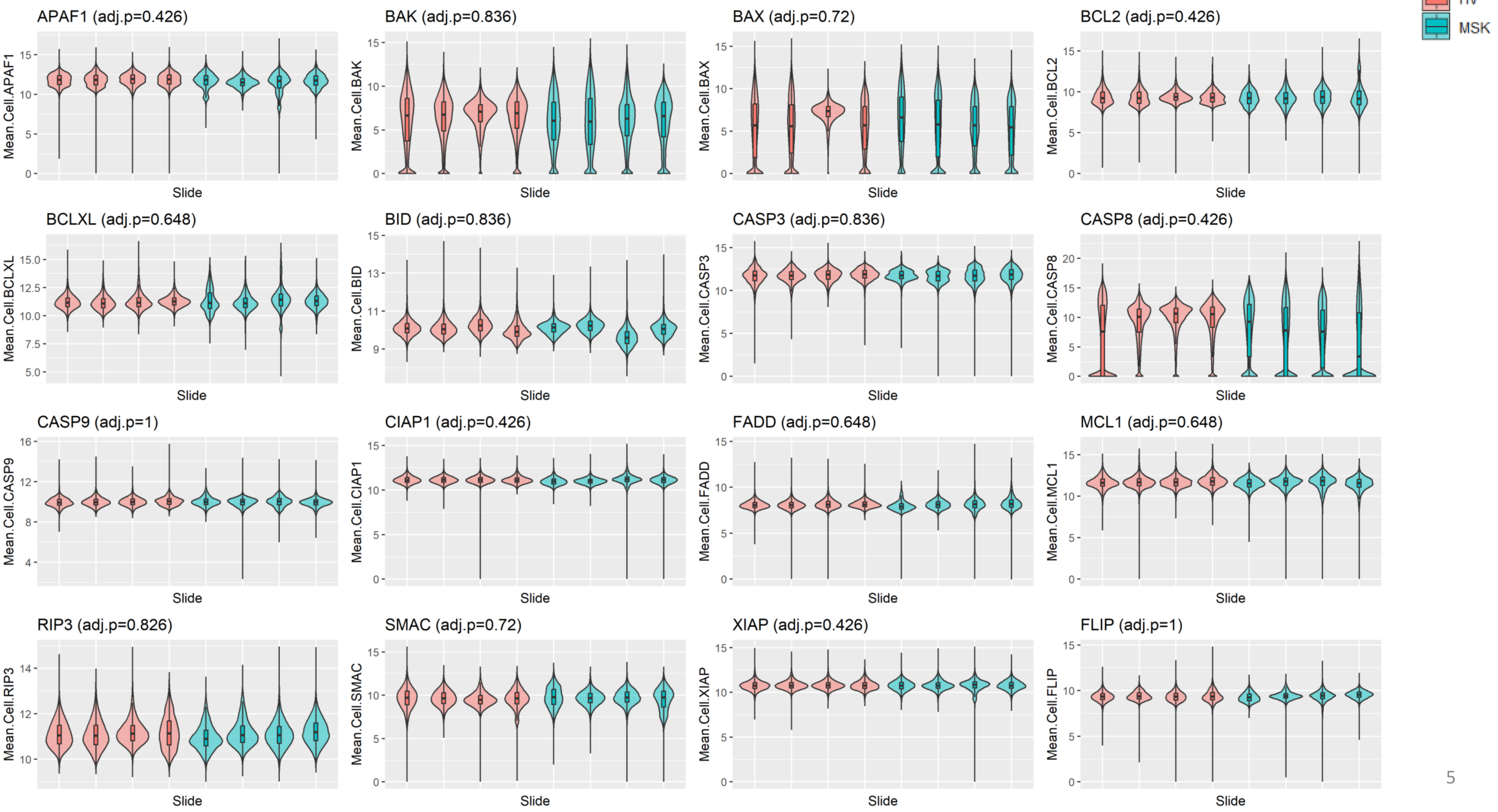

Supplementary Figure 5 a Correlation analysis between all apoptosis markers based on average intensity in the epithelial region. Color, ellipse shape and the numerical values correspond to the estimated Spearman correlation; **b** Hazard ratio is based on recurrence within 5 years between high vs. low (median cut-off) in average intensity of each biomarker. Lines represents 95% confidence interval of HR. None of the markers were significant after applying multiple hypothesis testing correction using BH method (FDR < 0.2)

a

b

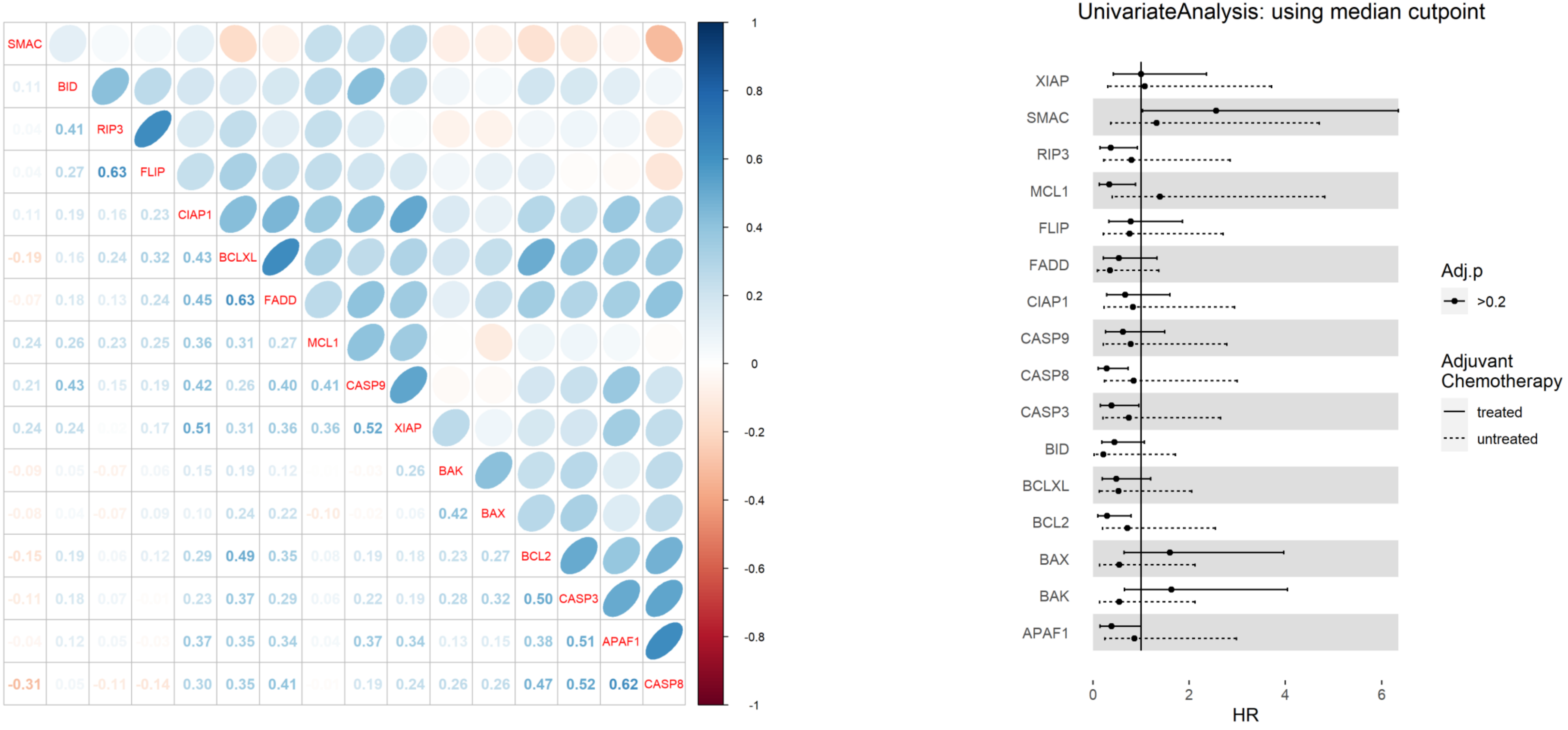

Supplementary Figure 6 – UMAP distributions of clusters illustrating distinct cell clusters for 1, 3 and 4 and overlaps for cluster 2, 5 and 6.

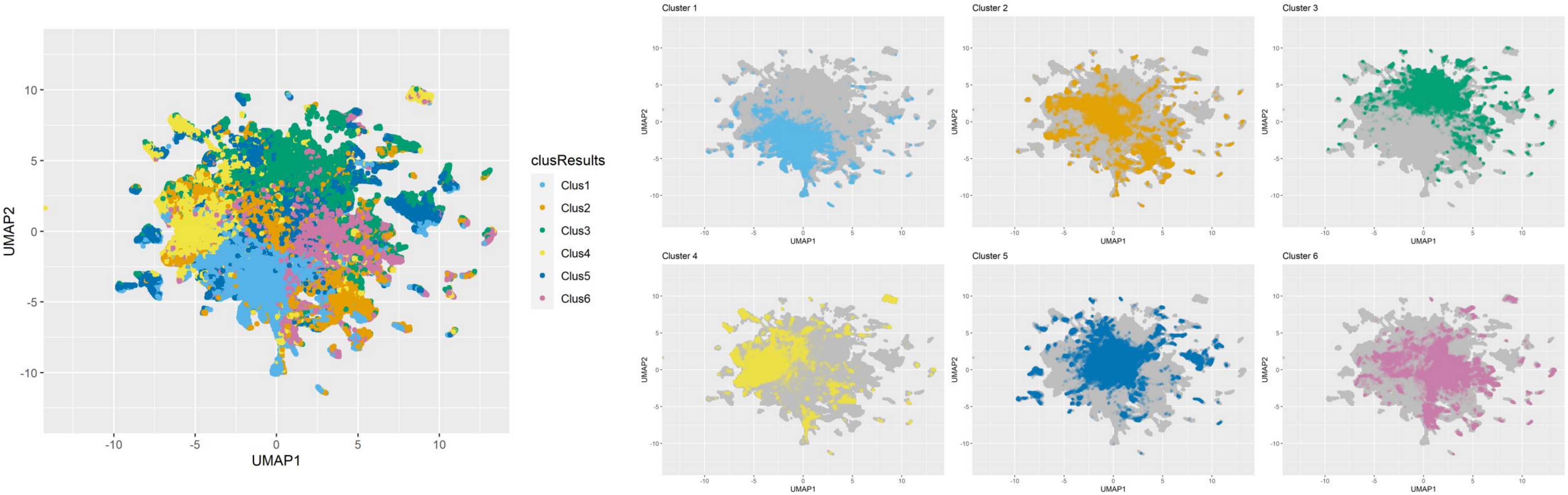

Supplementary Figure 7: Intrinsic pathway clusters and recurrence risk: To further interrogate the findings from the integrated pathway analysis, K-means clustering was done for the intrinsic pathway proteins using HV and MSK1 cohorts; **a** Heat map of clusters in the intrinsic pathway in HV and MSK1; **b** Multivariate model for cluster 2 and 4 in treated patients only adjusted for age and sex. Both cluster 2 and 4 were significant; **c-d** Kaplan Meier plots for adjuvant chemotherapy treated patients showing higher % cluster 2 (“caspase 3 inhibited”) and lower % cluster 4 (“caspase 9 primed”) are associated with increased recurrence risk; **e-f** no significant associations were found in surgery-only patients

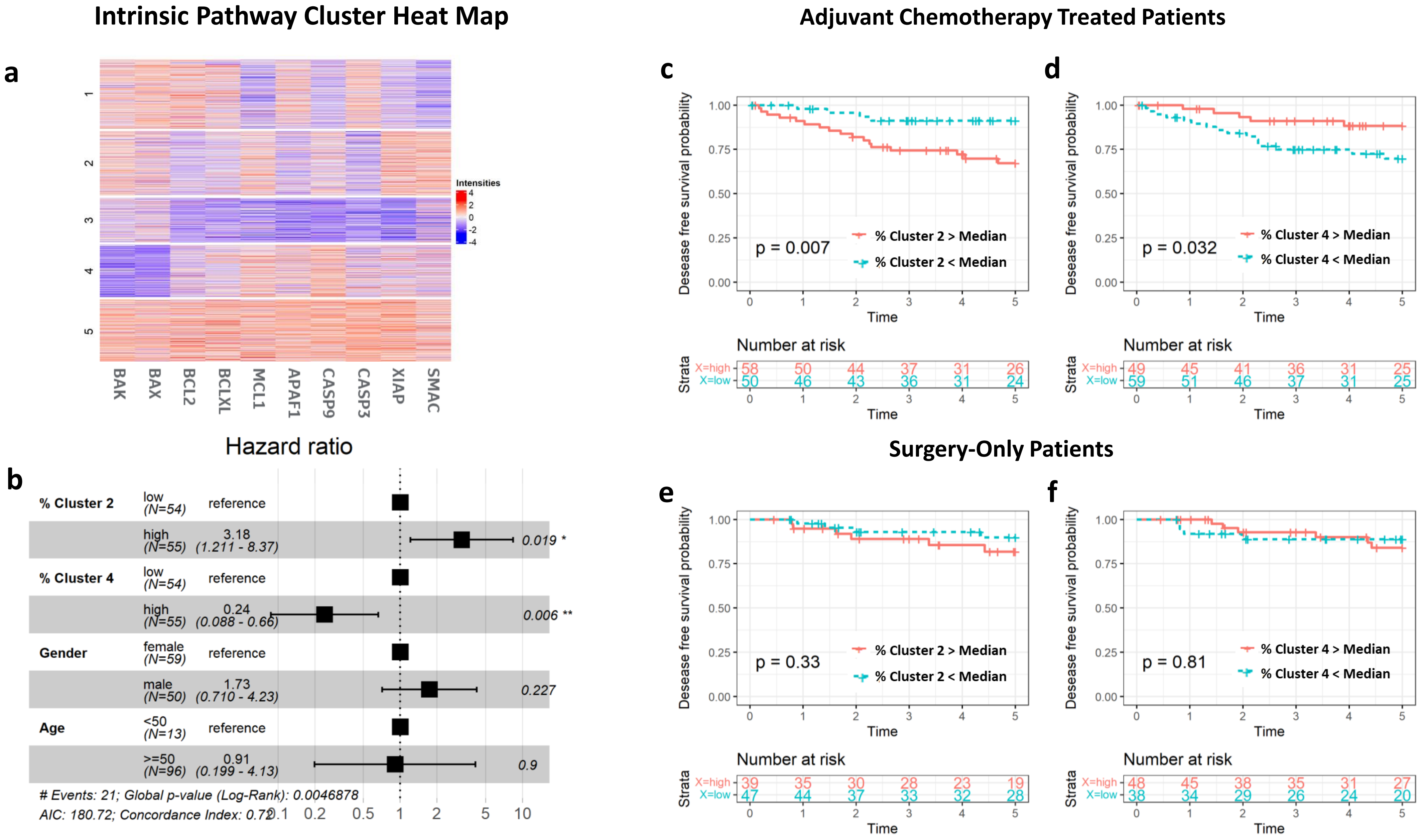

Supplementary Figure 8a - Flow-down chart for each step of clinical data filtering, including clinical parameters, data QC for the MSK2 stage II validation cohort (*n*=91); **b –** single cell data QC and filtering

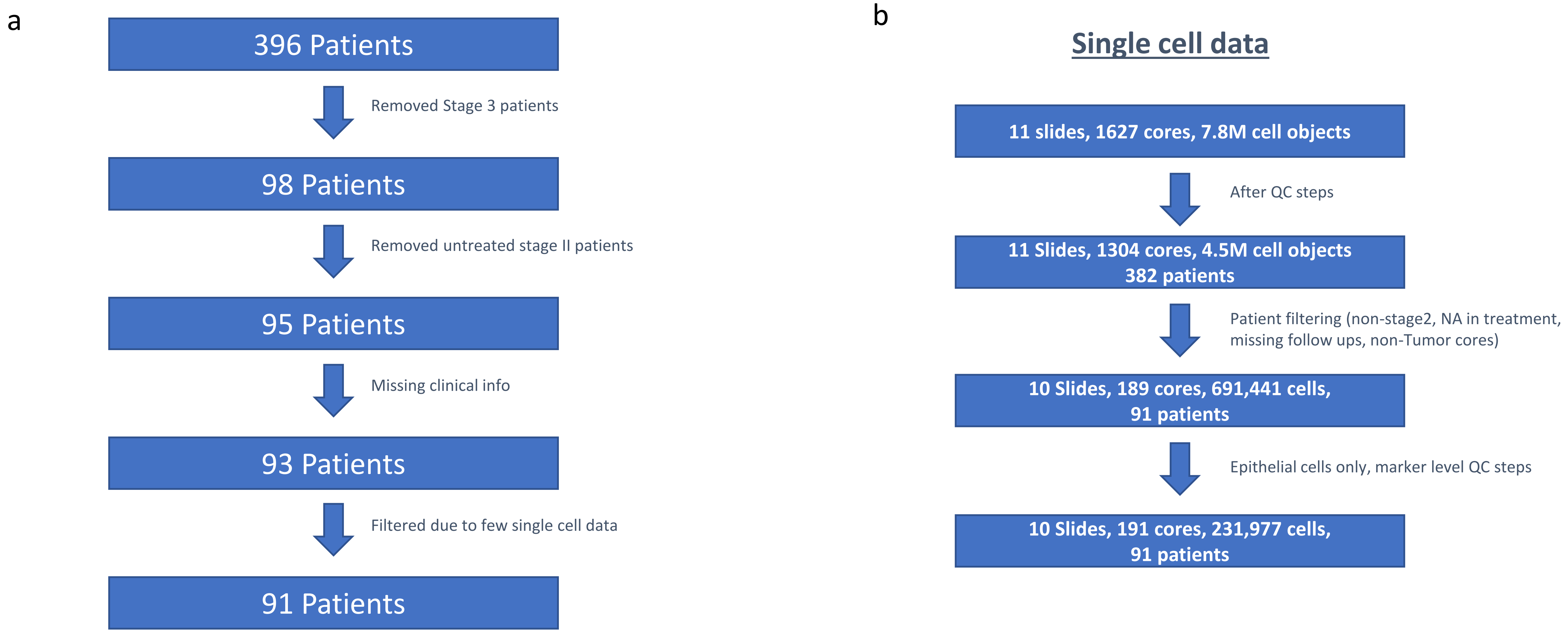

Supplementary Figure 9: Correlation between cluster profile and clinical factors. To further investigate the correlations between cluster profiles (a. cluster 2 and 4 for discovery cohort HV+MSK1 and cluster 2 for MSK2 (validation cohort) and clinical factors, we performed Spearman correlation test for continuous variables, and Wilcox/Kruskal test for categorical variables. Some clinical variables such as MMR, lymphovascular invasion, and local/distant metastasis were only available for MSK1 and MSK2; **a -** There was a weak trend for MMR-proficient patients to have either high % cluster 2 or high cluster 4 cells; **b – MSK2 -** Although there were no significant differences after adjustment for multiple testing, T4 cancers had a trend for higher % cluster 2 cells; female patients also had higher % cluster 2; **c –** We also evaluated associations between the cluster profile and local or distant metastases**.** Although non-significant after adjustment, MSK1 patients with higher % cluster 4 cells tended to have more local recurrence than distant metastasis; **d – MSK2 -** Cluster 2 was not associated with local or distant metastases.

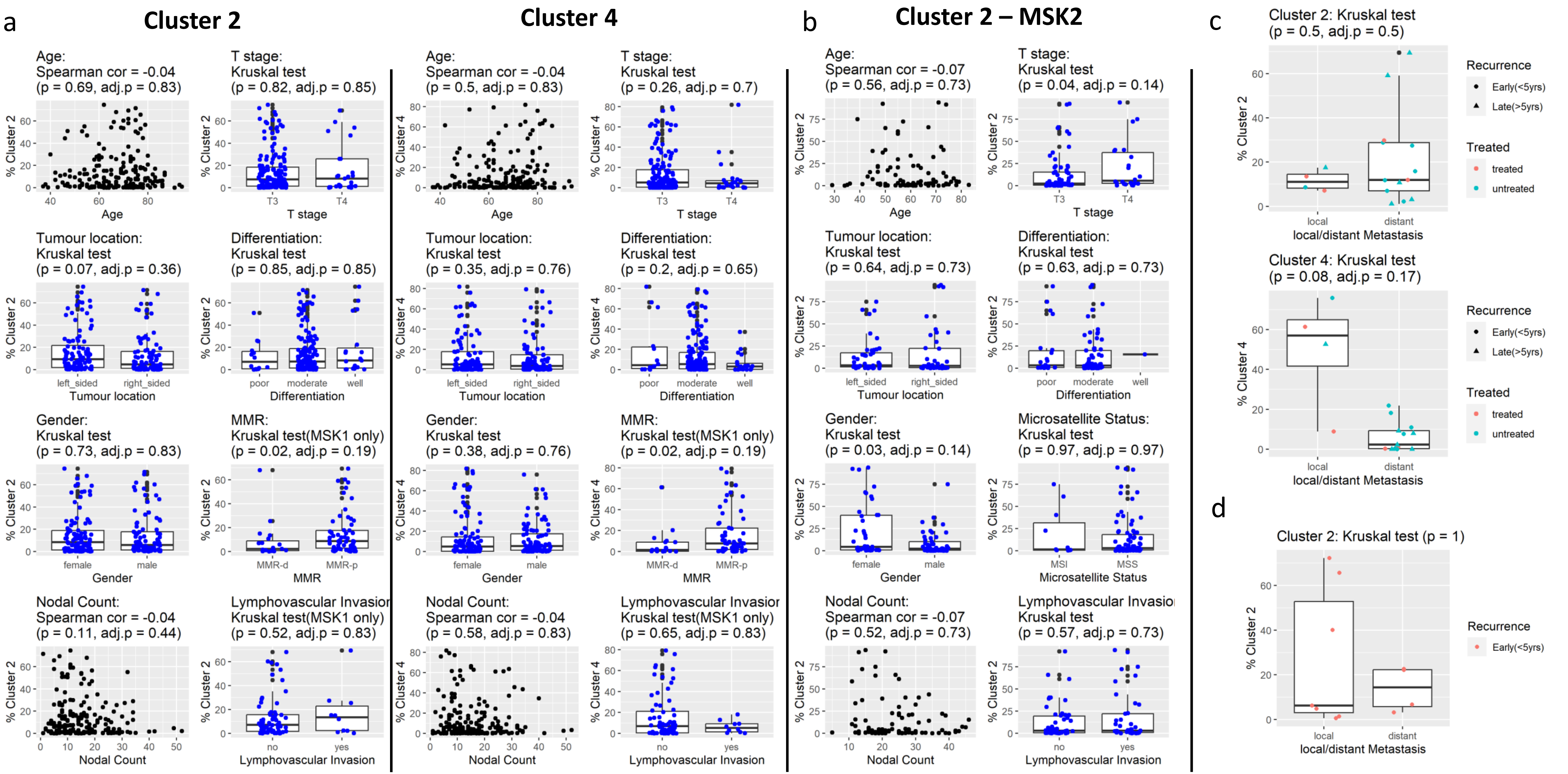

Supplementary Tables

Supplementary Table 1: Summary statistics for clinical parameters in treated and untreated patients from HV and MSK1 before filtering and after filtering, including excluded patients. There was no significant differences in the clinical parameters in treated and untreated patients after data filtering.

Before Filtering

	Untreated (N=282)	Treated (N=108)	Total (N=390)
**Age**			
Mean (SD)	69.798 (11.049)	64.343 (11.958)	68.287 (11.554)
Range	37.000 - 96.000	37.000 - 94.000	37.000 - 96.000
**Gender**			
female	131 (46.5%)	59 (54.6%)	190 (48.7%)
male	151 (53.5%)	49 (45.4%)	200 (51.3%)
**Differentiation**			
poor	17 (6.0%)	7 (6.5%)	24 (6.2%)
moderate	247 (87.6%)	88 (81.5%)	335 (85.9%)
well	18 (6.4%)	13 (12.0%)	31 (7.9%)
**Tumor location**			
left_sided	142 (50.4%)	52 (48.1%)	194 (49.7%)
right_sided	140 (49.6%)	56 (51.9%)	196 (50.3%)
**Nodal Count**			
Mean (SD)	15.936 (8.315)	15.843 (9.478)	15.910 (8.640)
Range	2.000 - 49.000	1.000 - 52.000	1.000 - 52.000
**T stage**			
T3	271 (96.1%)	94 (87.0%)	365 (93.6%)
T4	11 (3.9%)	14 (13.0%)	25 (6.4%)
**Follow up Time (Years)**			
Mean (SD)	6.120 (4.165)	5.070 (3.314)	5.829 (3.972)
Range	0.020 - 17.380	0.040 - 16.130	0.020 - 17.380
**Recurrence**			
no	244 (86.5%)	83 (76.9%)	327 (83.8%)
yes	38 (13.5%)	25 (23.1%)	63 (16.2%)

After Filtering

	Excluded (N=196)	Untreated (N=86)	Treated (N=108)
**Age**			
Mean (SD)	69.658 (11.076)	70.116 (11.044)	64.343 (11.958)
Range	37.000 - 96.000	39.000 - 93.000	37.000 - 94.000
**Gender**			
female	85 (43.4%)	46 (53.5%)	59 (54.6%)
male	111 (56.6%)	40 (46.5%)	49 (45.4%)
**Differentiation**			
poor	10 (5.1%)	7 (8.1%)	7 (6.5%)
moderate	174 (88.8%)	73 (84.9%)	88 (81.5%)
well	12 (6.1%)	6 (7.0%)	13 (12.0%)
**Tumor location**			
left_sided	90 (45.9%)	52 (60.5%)	52 (48.1%)
right_sided	106 (54.1%)	34 (39.5%)	56 (51.9%)
**Nodal Count**			
Mean (SD)	16.520 (8.189)	14.605 (8.494)	15.843 (9.478)
Range	2.000 - 41.000	2.000 - 49.000	1.000 - 52.000
**T stage**			
T3	196 (100.0%)	75 (87.2%)	94 (87.0%)
T4	0 (0.0%)	11 (12.8%)	14 (13.0%)
**Follow up Time (Years)**			
Mean (SD)	5.970 (4.023)	6.462 (4.477)	5.070 (3.314)
Range	0.020 - 15.530	0.450 - 17.380	0.040 - 16.130
**Recurrence**			
no	174 (88.8%)	70 (81.4%)	83 (76.9%)
yes	22 (11.2%)	16 (18.6%)	25 (23.1%)

Supplementary Table 2 – Antibodies for the apoptosis markers were included in this study. Clone, vendor details, fluorophore and staining concentrations are provided.

Target	Uniprot ID	Clone	Vendor	Catalog	Fluorophore	Staining Concentration (µg/mL)
Apaf-1	O14727	2E12	Millipore	MAB3503	Cy5	5
Bak	Q16611	Y164	Abcam	ab220790	Cy3	10
Bax	Q07812	E63	Abcam	ab216985	Cy3	10
Bcl-2	P10415	E17	Abcam	ab190577	Cy5	10
Bcl-XL	Q07817	7D9	Thermo	MS-1334	Cy5	10
BID	P55957	B-3	Santa Cruz	sc-373939	Cy5	5
BIM$_{L}$	O43521	polyclonal	R&D	AF1325	Cy3	5
Caspase-3 (Pro & cleaved)	P42574	D3R6Y	Cell Signaling	14214	Cy5	10
Procaspase-8	Q14790	EPR162	Abcam	ab108333	Cy3	5
Procaspase-9	P55211	96.1.23	Santa Cruz	sc-56076 AF647	Alexa647	5
cIAP-1	Q13490	polyclonal	R&D	AF8181	Cy5	5
FADD	Q13158	EPR5030	Abcam	ab229444	Cy3	10
FLIP	O15519	D5J1E	Cell Signaling	56343	Alexa555	7.5
MCL-1	Q07820	C-2	Santa Cruz	sc-7443	Cy3	5
RIP3	Q9Y572	B-2	Santa Cruz	sc-374639	Cy5	10
Smac	Q9NR28	79-1-83	Cell Signaling	2954	Cy5	5
XIAP (API3)	P98170	polyclonal	Thermo	APH937	Cy5	5
**Cell segmentation markers**						
Pan cytokeratin (1, 5, 6, 8)		CK26	Sigma	C5992	Cy3	2.5
Na+K+ATPase		EP1845Y	Abcam	ab167390	Cy3	5
Ribosomal S6		C-8	Santa Cruz	sc-74459	Alexa647	5

Supplementary Table 3: Clinical demographics for independent treated cohort (MSK2), with treated cohort (HV and MSK1) shown for reference. Range or patient number and % for each category are shown.

	MSK2 Treated Patients (n=91)	Treated Cohort 1 (HV and MSK1)(n=108)
**Age (years; mean, range)**	59.4 (29-83)	64.3 (37-94)
**Sex**		
**female**	42 (46.2%)	59 (54.6%)
**male**	49 (53.8%)	49 (45.4%)
**Tumor Differentiation**		
**poor**	22 (24.2%)	7 (6.5%)
**moderate**	68 (74.7%)	88 (81.5%)
**well**	1 (1.1%)	13 (12.0%)
**Tumor site**		
**Left**	45 (49.5%)	52 (48.1%)
**Right**	40 (44.0%)	56 (51.9%)
**undetermined_origin**	6 (6.6%)	
**Nodal count (mean, range)**	23.9 (5-46)	15.8 (1-52)
**T stage**		
**T3**	69 (75.8%)	94 (87.0%)
**T4**	22 (24.2%)	14 (13.0%)
**Perineural invasion**		
**No**	53 (58.2%)	NA
**Yes**	38 (41.8%)	NA
**Lymphovascular invasion**		
**No**	37 (40.7%)	NA
**Yes**	41 (45.1%)	NA
**Suspected**	13 (14.3%)	NA
